# Toward telemedical diagnostics—clinical evaluation of a robotic examination system for emergency patients

**DOI:** 10.1177/20552076231225084

**Published:** 2024-01-09

**Authors:** Maximilian Berlet, Jonas Fuchtmann, Roman Krumpholz, Abdeldjallil Naceri, Daniela Macari, Christoph Jähne-Schon, Sami Haddadin, Helmut Friess, Hubertus Feussner, Dirk Wilhelm

**Affiliations:** 1 Department of Surgery, University Hospital rechts der Isar, 9184 Technical University of Munich, Munich, Germany; 2 MITI Research Group, University Hospital rechts der Isar, 9184 Technical University of Munich, Munich, Germany; 3 Munich Institute of Robotics and Machine Intelligence, 9184 Technical University of Munich, Munich, Germany; 4 585210 Franka Emika GmbH, Munich, Germany; 5 Max Planck Institute for Intelligent Systems, 630599 Max Planck ETH Center for Learning Systems, Stuttgart, Germany

**Keywords:** telemedicine, clinical examination, robotics, future healthcare, emergency room, coronavirus

## Abstract

**Introduction:**

The SARS-CoV-2 pandemic has affected global public healthcare for several years. Numerous medical professionals have been infected since the outbreak in 2019, resulting in a shortage of healthcare providers. Since traditional personal protective wear was insufficient to eliminate the virus transmission reliably, new strategies to avoid cross-infection were imperative while enabling high-quality medical care. In the project ProteCT, we investigated the potential of robotic-assisted examination in providing medical examination via a telemedical approach.

**Material and Methods:**

We constructed a fully functional examination cabin equipped with cameras, microphones, screens and robotic arms to evaluate usability and perception. Therefore, we conducted a preliminary study with 10 healthy volunteers and 10 physicians to gain first insights and optimize the setup. In a second step, we performed telemedical examinations of actual patients from the local emergency department to compare the robotic approach with the classical method of measuring vital signs, auscultation, palpation and percussion.

**Results:**

The preliminary study identified basic requirements, such as the need for force-feedback and telemedical training for physicians. In the main study, acceptance was high and most patients indicated they would use a telemedical system again. Our setup enabled the physician to make the same diagnoses as by classic examination in the emergency department in most cases.

**Discussion:**

The potential acceptance of a telemedical system such as ProteCT is high. Robotic telemedical approaches could complement future healthcare beyond the Corona pandemic to reach rural areas or even war zones. Moreover, the daily clinical use of robotic telemedicine could improve patients’ safety, the quality of perioperative management and the workflow in any medical facility.

**Conclusion:**

The development of telemedical and telerobotic systems is a multidisciplinary and complex challenge. However, acceptance of the proposed system was high among patients and physicians, indicating the potential use of similar systems for future healthcare.

## Introduction

From 2020 to 2023, the Coronavirus 2 (SARS-CoV-2) has kept the world in suspense. Besides apparent adverse effects such as increased mortality and the Long COVID Syndrome, even healthcare sections without immediate relation to the pandemic have been affected.^1,2^ The World Health Organization's interim report about COVID-19, issued in August 2020, highlighted restricted access to inpatient and outpatient services, community-based care and mobile clinics. At least partial disruption of healthcare, even for non-communicable diseases, was reported by 69% of the countries included in the study. One often-mentioned issue was the patients’ fear of getting infected while visiting medical facilities. On the other side, insufficient personal protective equipment (PPE) for healthcare providers and a shortage of qualified clinical staff were responsible for the compromised medical supply, concluding that most national healthcare systems could not adequately manage the challenges dictated by the pandemic.^3,4^ Therefore, solutions to ensure high-quality healthcare services while addressing the patients’ fear of getting infected when they visit medical institutions were mandatory. Additionally, protecting healthcare workers was a critical issue as their frequent contact with potentially infectious patients tremendously increases the probability of infection.^5^ The primary cross-infection mechanism of SARS-CoV-2 is spreading airborne virus particles. Especially in patient waiting areas of medical facilities, a high concentration was detected in the ambient air during several studies.^6^ Despite PPE being the current standard in avoiding cross-infection, several drawbacks exist with this approach. PPE is expensive, often of poor or unknown quality and causes much garbage.^7–9^ In addition, its daily clinical use can interfere with medical care and natural communication between physicians and patients, as the correct application is time-consuming and facial expressions are partially covered.^10–14^

However, SARS-CoV-2 cross-infection can easily be avoided by spatially separating medical staff and patients. Thus, the demand for telemedical applications increased drastically during the pandemic. Nevertheless, current telemedical solutions are mostly limited to online consultation without the sufficient possibility for physical examination.^15,16^ In other fields of medicine, such as surgery, robotic-assisted systems increasingly bridge barriers.^17,18^ In the following article, we assess whether robotic tele-examination can be a valid option to prepare future healthcare systems when facing challenges like pandemics, shortages of medical professionals and general healthcare supply.

In this regard and focusing on general acceptance, a comprehensive robotic-assisted examination system named ProteCT, funded by the German Federal Ministry of Education and Research (BMBF), was developed. In the first step, we evaluated our prototype setup in a preliminary study involving physicians and healthy volunteers. As a second step, we performed a clinical study with actual patients from our local emergency department at the University Hospital Klinikum rechts der Isar in Munich. In the following article, we report the results of both studies and discuss their relevance for future healthcare.

## Material and methods

In the scope of the project ProteCT, we developed the prototype of a telemedical system, covering all relevant modalities for a comprehensive initial clinical examination required to assess emergency cases while physically separating patients and medical professionals.^19^

### Robotic examination cabin

**Figure 1.**
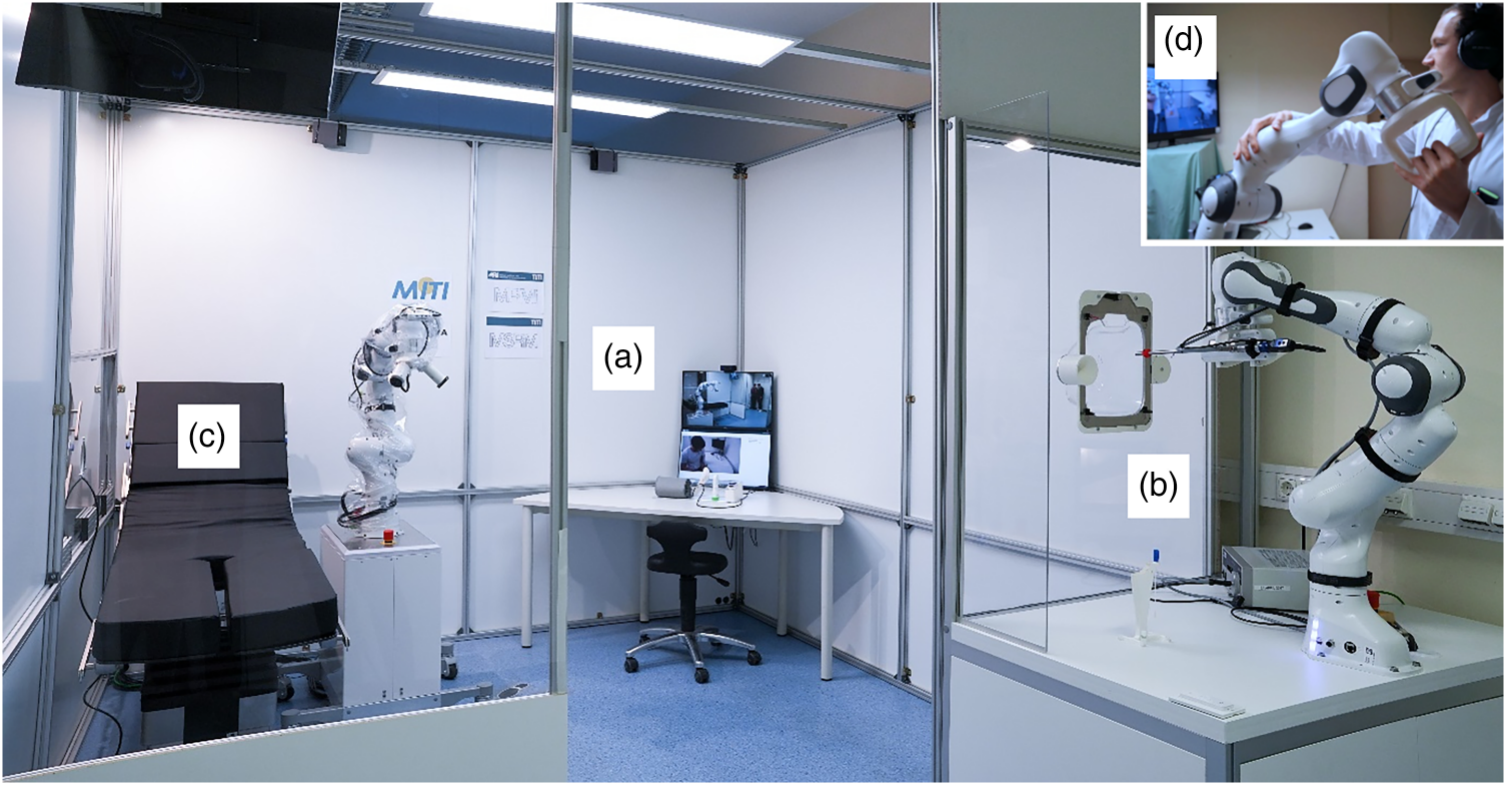
Telemedical examination cabin used within the studies. All captured diagnostic parameters are directly provided to a remote physician. The setup enables inter alia the check of vital signs, assessment of the chief complaints (a), a naso-pharyngeal swab, inspection of the oral cavity (b), auscultation, percussion, and palpation (c). The physician is situated in a remote cockpit and has control over all devices including the robotic arms inside the cabin (d).

Our telemedical setup consists of a patient examination cabin and a spatially separated control unit for the physician, which is installed in another room. The system is based on three modules inside the cabin, each providing a set of diagnostic tasks of similar technical complexity to allow for synchronous telemedical examination. Multiple monitors, cameras and microphones enable continuous real-time communication between physician and patient (Figure 1). In module **A**, the patient sits at a desk to obtain the chief complaints via video chat. Furthermore, parameters like blood oxygen saturation (Novidion GmbH, Cologne, Germany), temperature, heart rate and blood pressure (Withings France SA, Issy-les-Moulineaux, France) can be measured here. A self-developed camera-equipped otoscope for examination of the ears is also available. Short videos with instructions on each device are displayed on a screen during the examination process to supplement the physician's instructions. All devices transfer their results synchronously to the physician's graphical user interface (GUI) in the control unit distant from the cabin. The GUI was developed especially for this project. In module **B**, an automated nasopharyngeal swab enables testing for infection as well as a camera-based synchronous inspection of the oral cavity. The module makes use of a robotic arm (Franka Emika GmbH, Munich, Germany) that senses all applied forces and can interact on an impedance-controlled base, equipped with an endoscope (Karl Storz SE & Co. KG, Tuttlingen, Germany) and a fixture for a swab. An acrylic window with an exchangeable mask that provides two funnel-shaped openings for placing the patient's nose and mouth separates the robot and the patient. Apertures ensure a depth limitation for the automatic but patient-individual penetration of the oral and nasal cavity by the robotic end-effector. The physician can start the examination process over the GUI and interrupt it at anytime. The endoscope's video signal is transmitted synchronously to the GUI, allowing real-time reactions like advising the patient to say “A” for a better view. Module **C** consists of a similar robot (Franka Emika GmbH, Munich, Germany) but is placed freely within the cabin. A revolver-like fixture enables auscultation, percussion and palpation of the patient's chest and abdomen. The physician synchronously controls all movements using an identical lead robot in the distant control unit. The lead robot directly transfers modifications of its pose to the follower robot in the cabin in real-time, which is crucial regarding safety. Applied forces are transmitted in both directions, using force-feedback technology and allowing for a sensitive interaction and haptic experience. Finally, an on-site assistant can perform manual blood sampling through an acrylic hatch under safety precautions. Multiple high-end camera and microphone installations guarantee permanent bidirectional physician-to-patient and patient-to-physician communication during the patient's presence in the cabin as in a classic visit.

### Preliminary study

To evaluate the first functional prototype during the development process and to further optimize and improve the setup, we performed a preliminary study with 10 healthy volunteers playing predefined disease patterns as “actor patients” (Supplemental Material 9) and 10 medical doctors performing a standardized remote examination without prior training. The preliminary study was conducted in the same prototypical examination cabin as the later main study. We installed the entire system in the basement of the local emergency department of the University Hospital Klinikum rechts der Isar of the Technical University of Munich. The preliminary study was completed during the COVID-19 pandemic for three days in February 2021. After a short security instruction, each participating physician examined one actor patient spatially separated from each other, only using the telemedical system. Therefore, the system's control unit was installed in a room other than the patient cabin. Simulated laboratory results were presented to the physician individually for each disease pattern. At the end of the process, the physicians concluded the suspected diagnosis. An engineer was always available near the cabin for security reasons and kept written records of the whole process. Exclusion criteria for the healthy actor patients were pregnancy, underage and mental illness.

Questionnaires for all participants, examination protocols and the time log were the basis for evaluation (Supplemental Materials 1–3 and 6). The local ethical committee approved the study (No. 248/21 S-KH), and all actor patients and physicians gave written informed consent according to the Declaration of Helsinki. Moreover, we developed a hygiene concept tailored for SARS-CoV-2 in cooperation with the local clinical hygiene department for the preliminary and main study, which included disinfection of contact surfaces, a plan for room ventilation and the exchange of system parts having direct contact with the patient's body orifices (Supplemental Material 7). Of note, the final version of the robotic percussion adapter was yet to be available in the setup and the end effector for palpation did not yet provide force feedback during the preliminary study.

### Main study

With improvements based on the findings of the preliminary study and the comprehensive feedback regarding the medical functionality and safety perception of all participants, we conducted the main study with actual patients from the local emergency department for three days in the spring of 2021. It was completed in the same but substantially improved examination cabin as the preliminary study, installed in the basement of the emergency department at the University Hospital rechts der Isar of the Technical University of Munich. For this purpose, functional palpation force feedback, an improved auscultation adapter and a percussion end effector complemented the system. Moreover, the patient introduction procedure was adapted to improve the feeling of safety. Thus, each patient touched and pushed the robotic arm away once before the examination started to gain a feeling for the applied forces. A comprehensive risk assessment of all single components (Figure 1(a)–(d)) based on the ISO 12100:2010 allowed for the definition of safety criteria. During the COVID-19 pandemic, the cross-infection between patients and medical professionals was a major risk besides harming participants with the robot arms. As the primary intention of the whole system was a spatial separation of patients and physicians, virus transmission between these groups was excluded entirely. Moreover, the staff near the cabin wore protection masks, gloves and gowns and kept their distance from the participating patients. Rigorous implementation of the developed hygiene concept minimized the risk of cross-infection between subsequent patients. Regarding the physical perils in the examination cabin, we defined several safety precautions as a force-limitation in the robots, precise instructions with easy-to-understand icons, a design of all components that avoided sharp sites and emergency stop buttons reachable for participants and staff at all times. Standard emergency equipment and a physician were always available near the cabin, while the central emergency room was reachable within 1 min. The system was set up in the resuscitation team's area of responsibility and was reachable by them within a few minutes, equal to any other regular department in the hospital. Only one well-trained telephysician performed all telemedical examinations to reach a higher degree of routine and safety during the main trial.

**Figure 2.**
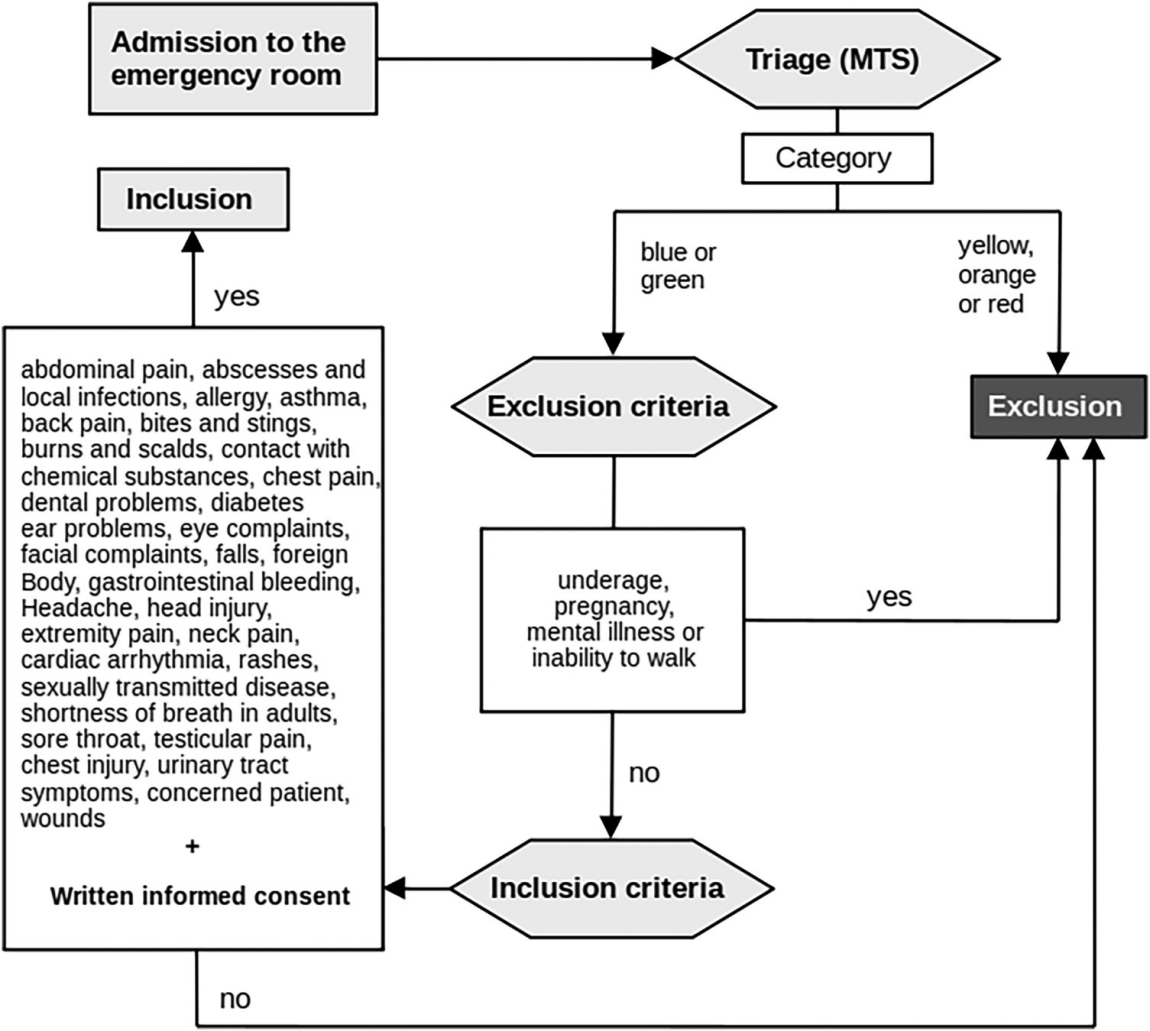
Inclusion algorithm of the ProteCT main study. MTS: Manchester triage system; application of the MTS leads to five possible triage categories with yellow, orange, and red being the most critical; blue or green classification indicates lower treatment priority.

For recruitment, emergency patients were asked randomly around the waiting area of the emergency department. None of them had had prior contact with the system. Only patients in stable conditions without urgent or critical medical issues were included. Hence, a Manchester Triage System (MTS) category higher than “green” or “blue” was an exclusion criterion to avoid delayed diagnostic or therapeutic steps. Pregnant women, children and patients with mental illness were also excluded (See Figure 2 for inclusion and exclusion criteria.) The study was approved accordingly by the local ethical committee at the Klinikum rechts der Isar University Hospital (No. 248/21 S-KH), and all patients gave their written informed consent following the Declaration of Helsinki. For consistency, all participants underwent the same structured, comprehensive examination procedure at all three stations regardless of their symptoms. The particular steps comprised an initial interview to obtain the chief complaints and the past medical history, general inspection, vital parameter measurements, otoscopy, robotic inspection of the oral cavity and comprehensive robotic auscultation, percussion and palpation of the thorax and abdomen. The procedure ended with a conclusive talk between the telephysician and the patient to discuss findings and subsequent therapy or further necessary diagnostics. An engineer near the cabin kept written records regarding the course of the examination, conducted a time log and documented potential adverse events. Finally, the patients evaluated the system by answering a survey based on the Telehealth Usability Questionnaire (TUQ) and further distinctive questions addressing our specific system^20^ (Supplemental material 5). The telephysician documented all his findings in a prepared and standardized form and concluded the suspected diagnosis (Supplemental material 6). The telemedical findings and the suspected diagnosis were then compared to the routine documentation by the emergency room physicians for each single case after therapy and discharge from the hospital.

## Results

### Preliminary study

The preliminary study was conducted without any harming of participants or adverse events that would have made an interruption of the examination process mandatory. Nevertheless, the need for several technical improvements became obvious. In its first version, the robot arm for examination sometimes stopped when the physician moved it too close to its range of motion borders. Another problem was a disturbing noise when using the auscultation adapter mounted to the robot arm caused by insufficient mechanical isolation. Some minor problems with the camera transmissions to the GUI required an extra screen during the preliminary study but could be fixed after short debugging and did not influence the planned course.

All included participants completed the telemedical examination and filled out the particular questionnaires. Supplemental material 8 lists the baseline characteristics of the preliminary study. Only 10% of the physicians have had prior contact with telemedical applications, while 40% of the healthy actor patients had used such applications before.

The healthy volunteers rated the pleasantness of the examination procedures in the cabin with an average of 3.6 to 4.3 on a five-point Likert scale, with 1 indicating the most unpleasant experience (Table 1). Thereby, an inspection of the oral cavity was considered the most uncomfortable of all procedures, with 3.6 ± 0.9 points. Regarding safety concerns, only 20% of the participants were worried before the examination. However, no participant was harmed, no adverse event leading to an interruption of the examination process occurred, and none of the actor patients canceled the study, 40% of the actor patients had safety concerns during the process. The participants emphasized that a more detailed introduction to the examination process would have been beneficial to reduce their worries during the examination. For example, prior physical contact with the robot arm could have improved their feeling of the maximum forces applied. Accordingly, the physical presence of medical assistant staff near the cabin was mentioned as beneficial for emergency interventions and general support (3.5 ± 1.4 points). The naturalness of interaction and conversation with the physician was rated with 3.5 ± 1.2 and 3.8 ± 1.1 points. Using systems like the proposed one was estimated to be highly reasonable in pandemic situations (4.9 ± 0.4) by the actor patients.

**Table 1.** Actor patients’ evaluation of the first prototypical examination cabin.

Parameter	Value			
**Worries regarding the system**	**%**			
Worried to be harmed by the system before examination	20			
Worried to be harmed by the system during examination	40			
Worried about replacement of human staff	10			
**Naturalness of the physician-patient contact**	**Mean**	**(SD)**	**Median**	**(IQR)**
Interaction with the physician via monitors	3.5	(1.2)	4.0	(1.3)
Naturalness of conversation via monitors and speakers	3.8	(1.1)	4.0	(1.0)
**Pleasantness of the examination process**	**Mean**	**(SD)**	**Median**	**(IQR)**
Interview about chief complaints and medical history	4.2	(0.7)	4.3	(0.9)
Inspection of the oral cavity at the swab station	3.6	(0.9)	3.5	(0.5)
Heart auscultation with prototype adapter	4.0	(0.8)	4.0	(0.3)
Lung auscultation with prototype adapter	4.3	(0.6)	4.5	(0.5)
Abdominal auscultation with prototype adapter	4.3	(0.4)	4.0	(0.4)
Abdominal palpation (without force feedback)	3.8	(0.9)	4.0	(1.0)
Otoscopy	4.2	(0.5)	4.3	(0.5)
**Assistant staff near the cabin**	**Mean**	**(SD)**	**Median**	**(IQR)**
Importance of staff near the cabin	3.5	(1.4)	4.0	(1.1)
Communication with the assistant staff	4.0	(0.7)	4.0	(1.0)
**Evaluation of the prototypic overall system**	**%**			
The current prototype system is ready for use in real patients without improvement	60			
	**Mean**	**(SD)**	**Median**	**(IQR)**
Reasonable in pandemic situations	4.9	(0.3)	5.0	(0.0)

Results of the preliminary study with n = 10 actor patients, SD**:** standard deviation, IQR: interquartile range, 5 points Likert's scale with “1” being worse and “5” being best. According to evaluation, four actor patients were concerned about their safety during the examination process, but there were no harmful or adverse events. The pre-examination instructions were then modified for the main study so that each patient touched the robotic arm before use and pushed it away once to gain a feel for the forces applied. Accordingly, the perception of safety was higher in the main study.

The physicians evaluated the technical approach of the end effectors and devices for vital parameter measurement with a mean of 2.6 to 4.7 points on a five-point Likert scale. The most significant critical aspects were the sound quality of the prototypic auscultation adapter (3.1 ± 1.1 points) and the lack of force feedback in the palpation concept (2.6 ± 0.9 points). The technical approach of the camera control and the devices for measurement of pulse, blood oxygenation and blood pressure were evaluated with 4.1 to 4.7 points on average.

The untrained physicians approved the examination robot's free steering by mirroring a leader-arm's movement as intuitive and easy to learn (4.0 ± 1.0 and 4.3 ± 0.7 points). Also, the learnability of the examination with the robotic arm at the swab station was estimated to be high. Nevertheless, most medical professionals emphasized that preliminary training with the robot and camera control would be mandatory for clinical use. They estimated using a system like ProteCT to be reasonable during a pandemic, with a mean of 4.2** **± 0.7 points.

The comparison of the telemedical examination approach with the classic face-to-face examination was one central key point of the project. Therefore, we defined the quality of the classic approach as the “0” point (“equal”) on a five-point Likert scale for quality assessment. An estimated quality of the telemedical approach, which is lower than the standard, would cause negative and improved quality positive values. The physicians judged that obtaining the chief complaints was almost as good as an in-person interview (−0.2 ± 0.4 points). The other examination modalities were considered inferior to the classic face-to-face contact. For example, inspection of the oral cavity was rated at −0.4 ± 1.2 points. However, the physicians pointed out that avoiding cross-infection by the robot-based examination outweighs this slight reduction in quality. The already-mentioned technical problems negatively influenced their evaluation of the other examination modalities. A disturbing noise induced the inferiority of the telemedical auscultation due to insufficient isolation from the robotic arm, and low quality in palpation resulted from the already mentioned lack of force feedback (Table 2). Nevertheless, 90% of the physicians assigned suspected diagnoses correctly after telemedically assessing the played disease patterns (Supplemental material 9).

**Table 2.** Physicians’ evaluation of the first prototypical examination cabin.

Parameter		Value			
Quality of the telemedical examination process compared with classic examination (0 = equal to classic examination)		Mean	(SD)	Median	(IQR)
Obtaining chief complaints	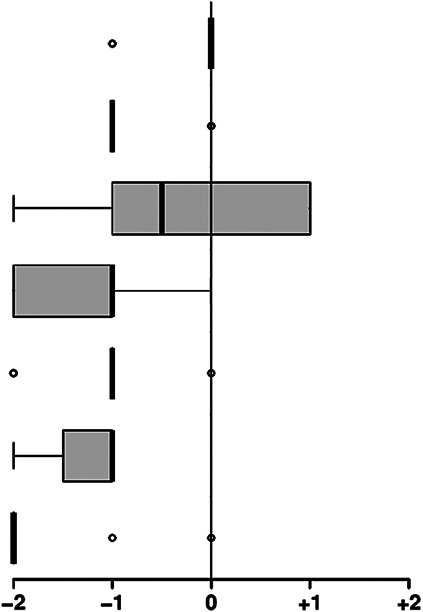	−0.2	(0.4)	0.0	(0.0)
General inspection via the system		−0.8	(0.4)	−1.0	(0.0)
Inspection of the oral cavity at the swab station		−0.4	(1.2)	−0.5	(1.8)
Heart auscultation with prototype adapter		−1.3	(0.7)	−1.0	(1.0)
Lung auscultation with prototype adapter		−1.1	(0.6)	−1.0	(0.0)
Abdominal auscultation with prototype adapter		−1.3	(0.5)	−1.0	(0.5)
Abdominal palpation (without force feedback)		−1.7	(0.7)	−2.0	(0.0)
**Technical approach of the system ProteCT**					
Camera control		4.1	(1.0)	4.0	(1.0)
End effector for auscultation		3.1	(1.1)	4.0	(2.0)
End effector for palpation (without force feedback)		2.6	(0.9)	3.0	(1.0)
Implementation of otoscopy		3.2	(1.2)	3.0	(1.0)
Implementation of pulse oximetry		4.7	(0.5)	5	(0.8)
Implementation of blood pressure measurement		4.6	(0.5)	5	(1.0)
**Technical approach of the examination arm**					
Intuitivity of robot control		4.0	(1.0)	4.0	(1.0)
Learnability of robot control		4.3	(0.7)	4.0	(1.0)
Security of robot control		4.1	(1.1)	4.0	(1.0)
Communication during examination		4.1	(0.6)	4.0	(0.0)
**Technical approach of the robot arm at the swab station**					
Intuitivity of robot control		4.0	(1.1)	4.0	(1.3)
Learnability of robot control		4.0	(1.0)	4.0	(2.0)
Security of robot control		4.0	(0.8)	4.0	(1.0)
Communication during examination		4.4	(0.5)	4.0	(1.0)
**Usefulness depending on availability of diagnostic options**					
Only percussion, auscultation, and palpation available		2.8	(1.4)	2.3	(2.6)
Only percussion, palpation, auscultation, vital parameter measurement, otoscopy, oral cavity inspection, and corona swab available		3.7	(0.7)	3.8	(1.3)
All diagnostic options including blood sampling available		4.4	0.3	4.5	(0.3)
**Evaluation of the prototypic overall system**		**%**			
The current prototype system is ready for use on real patients without improvement		60			
		**Mean**	**(SD)**	**Median**	**(IQR)**
Reasonable in pandemic situations		4.2	(0.7)	4.3	(0.5)

Results of the preliminary study with n = 10 physicians, SD: standard deviation, IQR: interquartile range, 5 points Likert’s scale with “1” being worse and “5” being best. The end effector for auscultation was improved for the main study by implementing better noise reduction, and force feedback was added to the end effector for palpation to allow perception of abdominal wall tension.

Consequently, in the optimization process, we addressed all deficiencies the healthy volunteers and physicians had mentioned. A new silicone coupling improved isolation between the auscultation adapter and the robotic arm, and a sufficient force feedback mechanism in the palpation approach was implemented. The fact that all medical professionals recommended preliminary training to ensure a safe examination process led to the decision that only one well-trained physician should perform all examinations and interviews in the main study with actual patients. Based on our experiences, a newly developed, structured training curriculum guaranteed complete control and safety of all functionalities. This concept comprises stepwise training exercises with patient models to improve dexterity and a theoretical part to increase knowledge about the technical background and security aspects.^21^

### Main study

**Figure 3.**
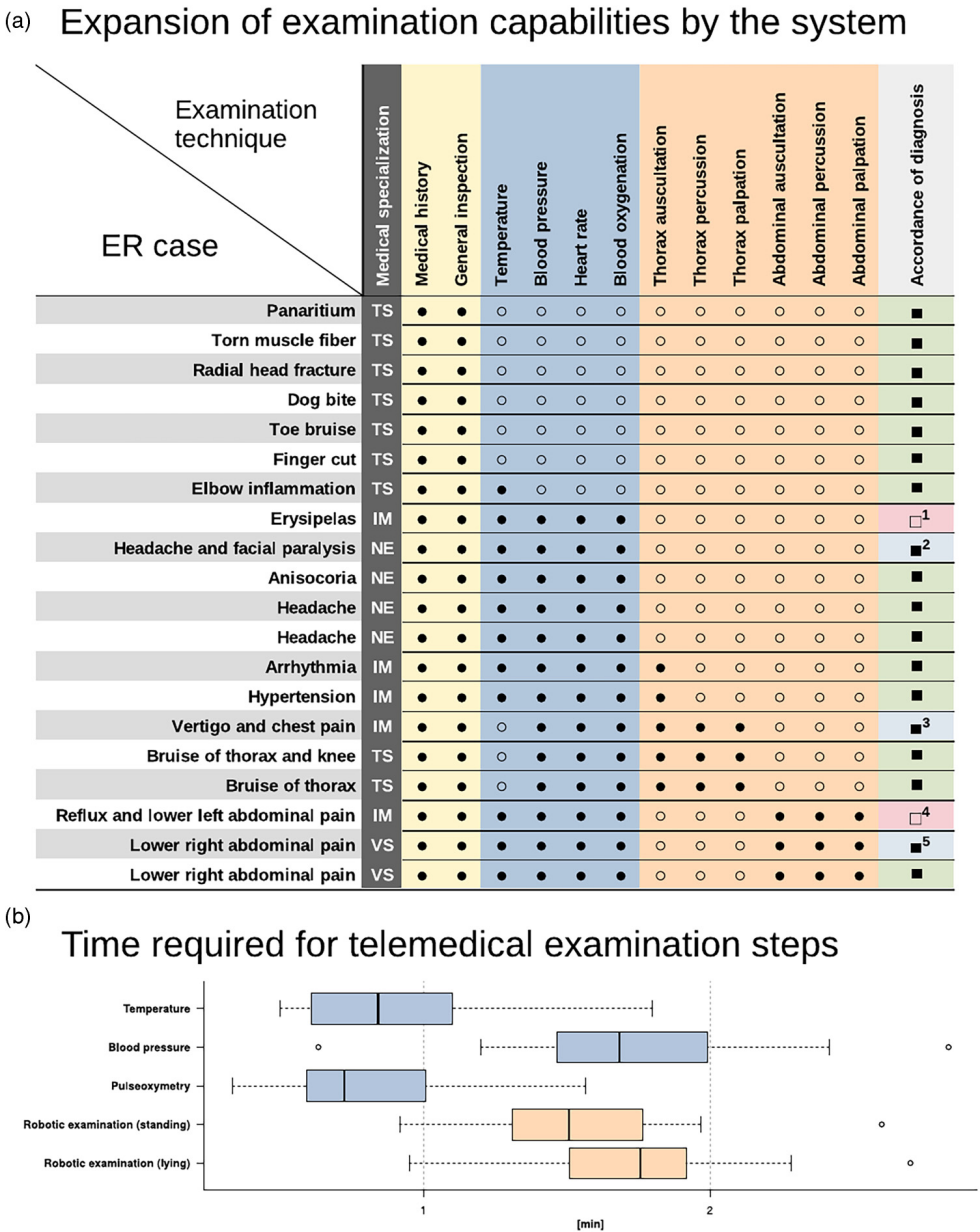
(a) Filled circles indicate the relevance of distinct examination techniques to find a diagnosis in the specific case. TS: Trauma surgery; IM: Internal medicine; NE: Neurology; VS: Visceral surgery; filled rectangles in the last column indicate accordance between the diagnosis achieved by the use of the telemedical system compared to classical examination in the emergency department, 1: instead of an erysipelas, an allergic rash was suspected, 2: facial paralysis was not mentioned in the emergency center documentation but was present during examination in the study, 3: chest pain was assessed by laboratory tests and an acute coronary syndrome was excluded in the emergency center, 4: in the emergency center, a beginning sigmoid diverticulitis was suspected, but during the examination in the cabin the lower left abdominal pain was not triggered by palpation, 5: at a second follow-up, an appendicitis was excluded in the emergency center, but the first diagnosis was suspected appendicitis as well. (b) Median duration of particular examination steps; Examination of the oral cavity is not mentioned because it required 1 min in each case. Otoscopy is not mentioned in the figure because it is no standard examination modality for all patients in the emergency room. There were no relevant findings in the study by the use of telemedical otoscopy.

In total, 20 patients from the local emergency department participated in the main trial (7 female and 13 male). The mean age was 35 ± 12 years. Regarding medical sub-categories, 9 patients were assigned to trauma surgery (TS), 5 to internal medicine (IM), 4 to neurology (NE) and 2 to visceral surgery (VS). Three patients declined participation after comprehensively clarifying the system and the planned study. As an indicator of their communication behavior, the patients’ daily use of smartphones and computers were baseline items of the evaluation questionnaire. They all stated to use such devices in their private context, while 85% employ modern communication technologies at work. Only 4 patients have had prior contact with telemedicine or telediagnostics before (Supplemental material 10). No adverse events occurred during the study, and no patient was harmed. The median duration of the entire physician-patient contact was 15 min (IQR 4 min), with the most extended phase being the assessment of chief complaints in conjunction with measurements of vital parameters (median 7 min 30 s, IQR 1 min 30 s). The highly standardized oral cavity examination took precisely 1 min in all cases. Examination with the robotic arm at station **C** (auscultation, percussion and palpation) took 5 min in median (IQR 30 s). The conclusive interview between physician and patient took 1 min in median (IQR 1 min). The duration of distinct examination procedures is depicted in Figure 3(b).

Subsequently, we analyzed which techniques were necessary to make a diagnosis in every single case (Figure 3(a)). In this regard, for 8 of the TS patients, obtaining the chief complaints and a general inspection alone was sufficient. One patient with an elbow infection required additional measurement of temperature. All cases assigned to the neurological, internal medicine and visceral surgery departments required complete co-assessment of vital parameters. For the comprehensive evaluation of 8 cases (4 IM, 2 TS and 2 VS), examination with the robotic arm at station **C** was necessary to make a reliable diagnosis. For instance, heart auscultation excluded a significant cardiac valve dysfunction in one case of arrhythmia, hypertension and vertigo combined with chest pain. In two cases of thorax injury, a crepitation or an attenuated breathing sound, for example, caused by pleural effusion, could be excluded. The assessment of 3 patients with abdominal pain required a complete abdominal examination comprising auscultation, percussion and palpation with robotic force feedback. Thereby, acute appendicitis was present in one case and the patient underwent an emergency appendectomy the same day.

In 18 cases, the physicians in the emergency department and the tele-physician found the same diagnosis. In one case, an allergic rash was diagnosed instead of erysipelas. In the other case, lower left abdominal pain was not trigger-able in the palpation procedure as it was recently in the emergency room (Figure 3(a), last column). In the questionnaire aimed at pleasantness, the feeling of safety and easiness during the examination at the three stages **A-C**, participants rated the system as appropriate in its current version (Table 3). The TUQ results for the presented system are depicted in Table 4. Participants generally estimated telemedicine to be useful in facilitating access to the healthcare system (4.2 ± 0.8 points). Although the patients stated that telemedicine could not meet all their medical needs (3.4 ± 1.5 points), most would use telehealth systems again (4.4 ± 1.1 points).

**Table 3.** Evaluation questionnaire specifically for the final ProteCT system.

Item	Evaluation			
Pleasantness	Mean	(SD)	Median	(IQR)
The examination with the devices at the conversation table was pleasant	4.9	(0.3)	5.0	(0.0)
The examination with the equipment at the swab station was pleasant	4.5	(0.7)	5.0	(1.0)
The examination with the devices on the robotic arm in the cabin was pleasant	4.7	(0.4)	5.0	(1.0)
**Perception of safety**				
I felt safe during the examination with the equipment at the conversation desk	4.8	(0.3)	5.0	(0.0)
I felt safe during the examinations with the equipment at the swab station	4.3	(0.7)	4.0	(1.0)
I felt safe during the examinations with the equipment on the robotic arm in the cabin	4.8	(0.3)	5.0	(0.0)
**Easyness**				
Performing the examinations at the conversation desk was easy for me	4.6	(0.7)	5.0	(1.0)
Performing the examinations at the swab station was easy for me	4.9	(0.3)	5.0	(0.0)
Undergoing the examinations on the robotic arm in the cabin was easy for me	4.9	(0.3)	5.0	(0.0)

Results of the main study with n = 20 emergency patients, SD**:** standard deviation, IQR: interquartile range, 5 points Likert's scale with “1” being worse and “5” being best.

**Table 4.** Evaluation of the final ProteCT system using the telehealth usability questionnaire (TUQ).

Item	Evaluation			
Usefulness	Mean	(SD)	Median	(IQR)
Telehealth improves my access to healthcare services	4.2	(0.8)	4.0	(1.0)
Telehealth saves me time traveling to a hospital or specialist clinic	4.4	(0.7)	5.0	(1.0)
Telehealth provides for my healthcare needs	3.4	(1.5)	3.0	(2.5)
**Ease of use and learnability**				
It was simple to use this system	4.8	(0.3)	5.0	(0.0)
It was easy to learn to use the system	4.8	(0.4)	5.0	(0.3)
I believe I could become productive quickly using this system	4.5	(1.2)	5.0	(1.0)
**Interface quality**				
The way I interact with this system is pleasant	4.6	(0.5)	5.0	(1.0)
I like using the system	4.5	(0.5)	4.0	(1.0)
The system is simple and easy to understand	4.9	(0.4)	5.0	(0.0)
This system is able to do everything I would want it to be able to do	3.9	(1.1)	4.0	(2.0)
**Interaction quality**				
I could easily talk to the clinician using the telehealth system	4.8	(0.5)	5.0	(0.3)
I could hear the clinician clearly using the telehealth system	4.9	(0.3)	5.0	(0.0)
I felt I was able to express myself effectively	4.8	(0.4)	5.0	(0.3)
Using the telehealth system, I could see the clinician as well as if we met in person	4.5	(0.7)	5.0	(1.0)
**Reliability**				
I think the visits provided over the telehealth system are the same as in-person visits	4.2	(0.9)	4.5	(2.0)
Whenever I made a mistake using the system, I could recover easily and quickly	4.7	(0.4)	5.0	(1.0)
The system gave error messages that clearly told me how to fix problems	4.9	(0.3)	5.0	(0.0)
**Satisfaction and future use**				
I feel comfortable communicating with the clinician using the telehealth system	4.7	(0.6)	5.0	(1.0)
Telehealth is an acceptable way to receive healthcare services	4.4	(0.8)	4.5	(1.0)
I would use telehealth services again	4.4	(1.1)	5.0	(1.0)
Overall, I am satisfied with this telehealth system	4.6	(0.5)	5.0	(1.0)

Results of the main study with n = 20 emergency patients, SD**:** standard deviation, IQR: interquartile range, 5 points Likert's scale with “1” being worse and “5” being best

## Discussion

In this article, we propose a complete setup for clinical examination to evaluate robotic telemedicine, gathering feedback from physicians and patients to deduce general acceptance and usability in a clinical setting. Our robotic-assisted cabine allows us to complete various diagnostic tasks remotely, thereby preventing a SARS-CoV-2 cross-infection. Despite patients and medical staff being spatially separated, the possibility of physical interaction is maintained, thus providing extended examination options. Our main study compared the examination results of standard medical assessments in our local emergency department with those of a telephysician leveraging the telerobotic approach. Although the system currently provides limited functions, we proved that even an early robotic setup could achieve a similar diagnostic yield as traditional clinical in-person examination and a high degree of acceptance in emergency patients.

Apart from the prerequisites of purely medical capability, acceptance of robotics and telemedicine is essential when intending to realize a similar diagnostic system in a productive clinical setting. In this regard, we found positive perceptions among the participating volunteers, patients and physicians during our two studies, indicating that telemedical and robotic applications could play a more central role in future clinical routines. Our main study with actual patients revealed similar and reasonable time requirements regarding the telemedical process. Minor deviations in finding the final diagnosis were not related to the setup but to the patient's current condition and a slightly different conclusion from the examining physicians. These deviations would not have significantly impacted the resulting medical procedures, as further diagnostic and therapeutic steps were the same.

However, we must mention a few inevitable limitations of the study design. Though patients were randomly acquired in the emergency room, three potential participants who were skeptical about the robotic technology declined to participate. Therefore, the presented results may differ from the overall public perception that could have been obtained by including a wholly unselected collective. The average age of 35 ± 12 years may not reflect the whole population and conceptional adaption might be imperative if elderly or younger patients would be considered for examination in our telerobotic cabin. More refined approaches regarding the simplicity of communication and procedures would be necessary. However, the age group included in the study will be part of the future elderly generation, when such systems could become medical standards and their high acceptance of our approach may predict future acceptance even in elderly patients due to their higher affinity to technology, as they already use electronic communication extensively (Supplement 10).

Of note, during the examination process, participants felt safer and more comfortable if they were encouraged by the remote physician to touch and move the robotic examination arm themselves, thereby getting a feeling for the forces involved. Taking such psychological aspects into account could prevent a feeling of insecurity regarding future clinical applications. As indicated in Figure 3(a), all incorporated examination functions were required to make a diagnosis in 40% of the cases. However, additional tools, such as an ultrasound probe, would have been beneficial. Due to the modular concept of our prototype, mounting such a device to the robotic examination arm would be a small effort.^22^

In this sense and considering the performance of the entire ProteCT system, the current configuration must be further optimized and adapted to particular clinical questions and specialties. For instance, neurological and orthopedic examination capabilities, which still need to be implemented, could be realized with only minor modifications. However, even in developed countries, high initial costs, thorough maintenance and the demand for high-quality communication infrastructure are limiting factors. Especially if intending to bridge large distances by telemedicine, the interconnection of the components only by ethernet would not be practical. New data transmission technologies like 5G or subsequent standards are mandatory to make telemedicine flexible and widely available. Thus, even data-intensive modalities like telemedical real-time sonography could be implemented in more comprehensive telemedical and telerobotic solutions.^23,24^

Despite these challenges, the positive feedback that our setup received suggests a potential future application of robotic systems in the diagnostic field of telemedicine. While robotic examination can provide safe healthcare access during pandemics such as SARS-CoV-2, its possibilities expand further. Medical coverage of rural and remote regions or even war zones could be facilitated. Moreover, telemedical systems could be integrated into the daily clinical workflow at any medical facility to protect immune-compromised patients, support medical staff and improve perioperative care.^25,26^

Further expansion could path completely autonomous systems to serve as a “first responder” in the healthcare system, thus enabling an effective preselection and distribution to the best-fitting medical specialty.^27^ A strategic expansion, leading to a robotic telemedical backbone for the healthcare system, could improve equal access to high-quality medical services around an entire country and even globally. While our telemedical system is currently one of a kind, our findings confirm that fast-paced developments in robotic technology will benefit humanity by fostering comprehensive and effective healthcare.^28^ All the examples indicate the importance of further research in telemedicine and telediagnostics to strengthen global healthcare for future challenges.

## Conclusion

The development of comprehensive telemedical and telerobotic applications is complex and requires the consideration of logistic, technical, psychological and medical aspects. Accepted solutions require the collaboration of multiple disciplines, such as engineers, computer scientists and physicians. Stagewise evaluation with healthy volunteers and actual patients is mandatory to estimate future acceptance of a particular system. In our studies, the acceptance of telemedical and telerobotic applications was high in emergency patients. Thus, systems like the one proposed could improve future healthcare significantly.

## Supplemental Material

sj-docx-1-dhj-10.1177_20552076231225084 - Supplemental material for Toward telemedical diagnostics—clinical evaluation of a robotic examination system for emergency patients Click here for additional data file. Supplemental material, sj-docx-1-dhj-10.1177_20552076231225084 for Toward telemedical diagnostics—clinical evaluation of a robotic examination system for emergency patients by Maximilian Berlet, Jonas Fuchtmann, Roman Krumpholz, Abdeldjallil Naceri, Daniela Macari, Christoph Jähne-Schon, Sami Haddadin, Helmut Friess, Hubertus Feussner and Dirk Wilhelm in DIGITAL HEALTH
