# Supplementary material for: Toward telemedical diagnostics—clinical evaluation of a robotic examination system for emergency patients
Source: Digit Health. 2024 Jan 9;10:20552076231225084. doi: 10.1177/20552076231225084 (PMC10777806; doi:10.1177/20552076231225084)
Supplement: sj-docx-1-dhj-10.1177_20552076231225084 - Supplemental material for Toward telemedical diagnostics—clinical evaluation of a robotic examination system for emergency patients [file sj-docx-1-dhj-10.1177_20552076231225084.docx]

**Supplementary to the article**

**“Toward Telemedical Diagnostics — Clinical Evaluation of a Robotic Examination System for Emergency Patients”**

**Index of supplements**

**Supp. 1:** Protocol for the preliminary ProteCT study

**Supp. 2:** Questionnaire for actor patients (preliminary study)

**Supp. 3:** Questionnaire for physicians (preliminary study)

**Supp. 4:** Protocol for the ProteCT main study

**Supp. 5:** Questionnaire for patients (ProteCT main study)

**Supp. 6:** Medical Documentation Form for physicians

**Supp. 7:** Hygiene concept for the project ProteCT

**Supp. 8:** Baseline characteristics of participants of the preliminary ProteCT study

**Supp. 9:** Case examples of the preliminary ProteCT study

**Supp. 10:** Baseline characteristics of patients participating in the ProteCT main study


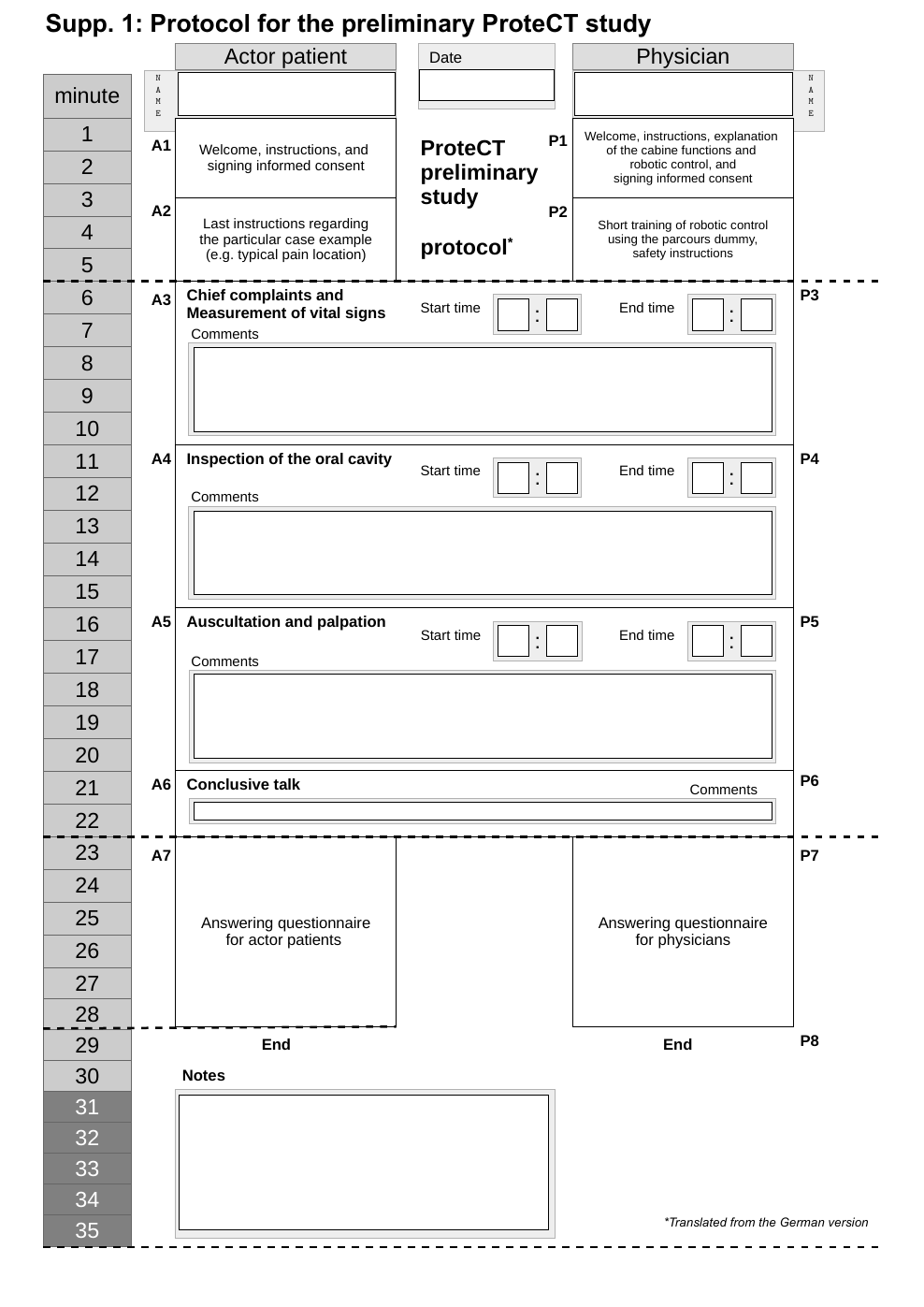


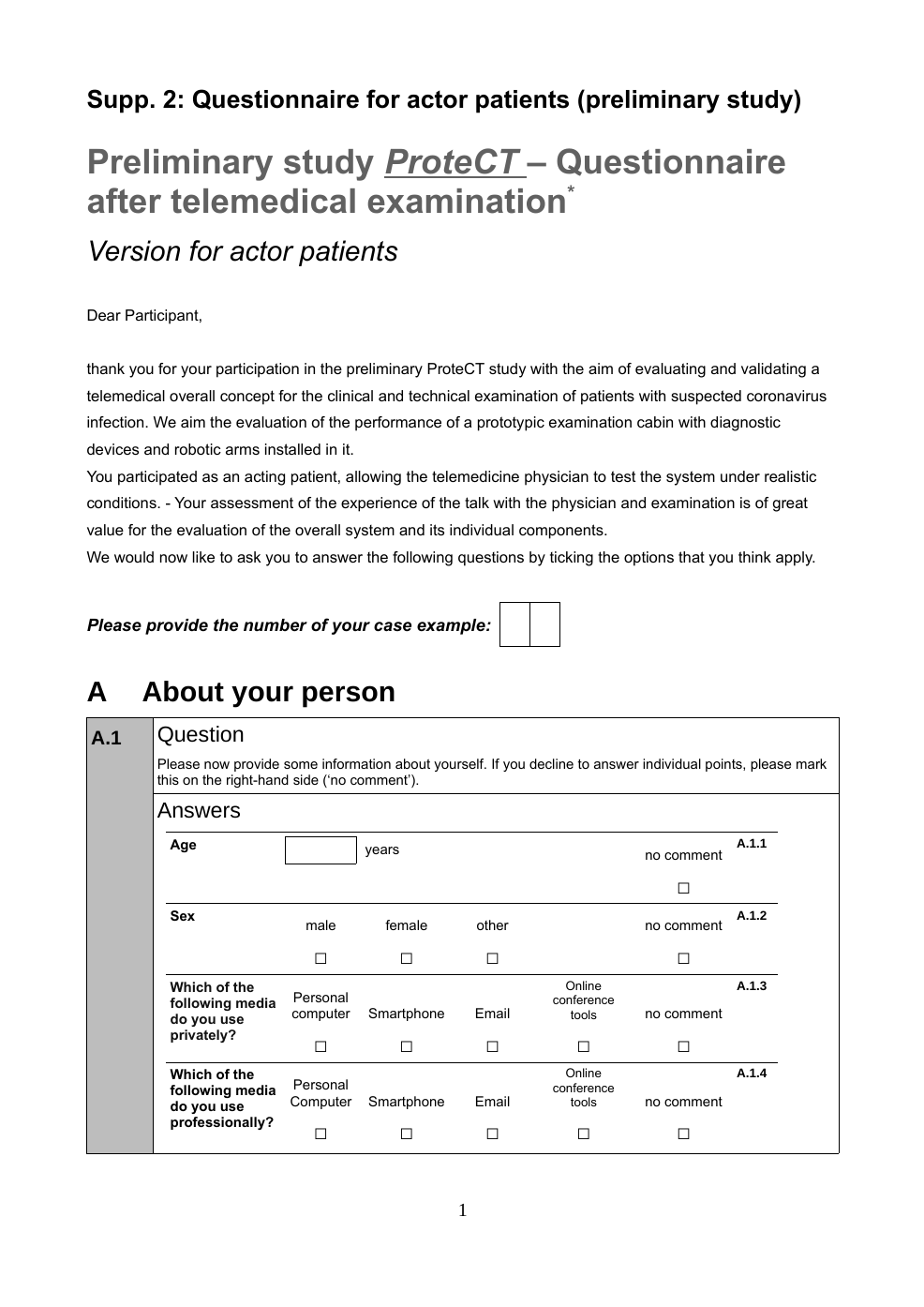


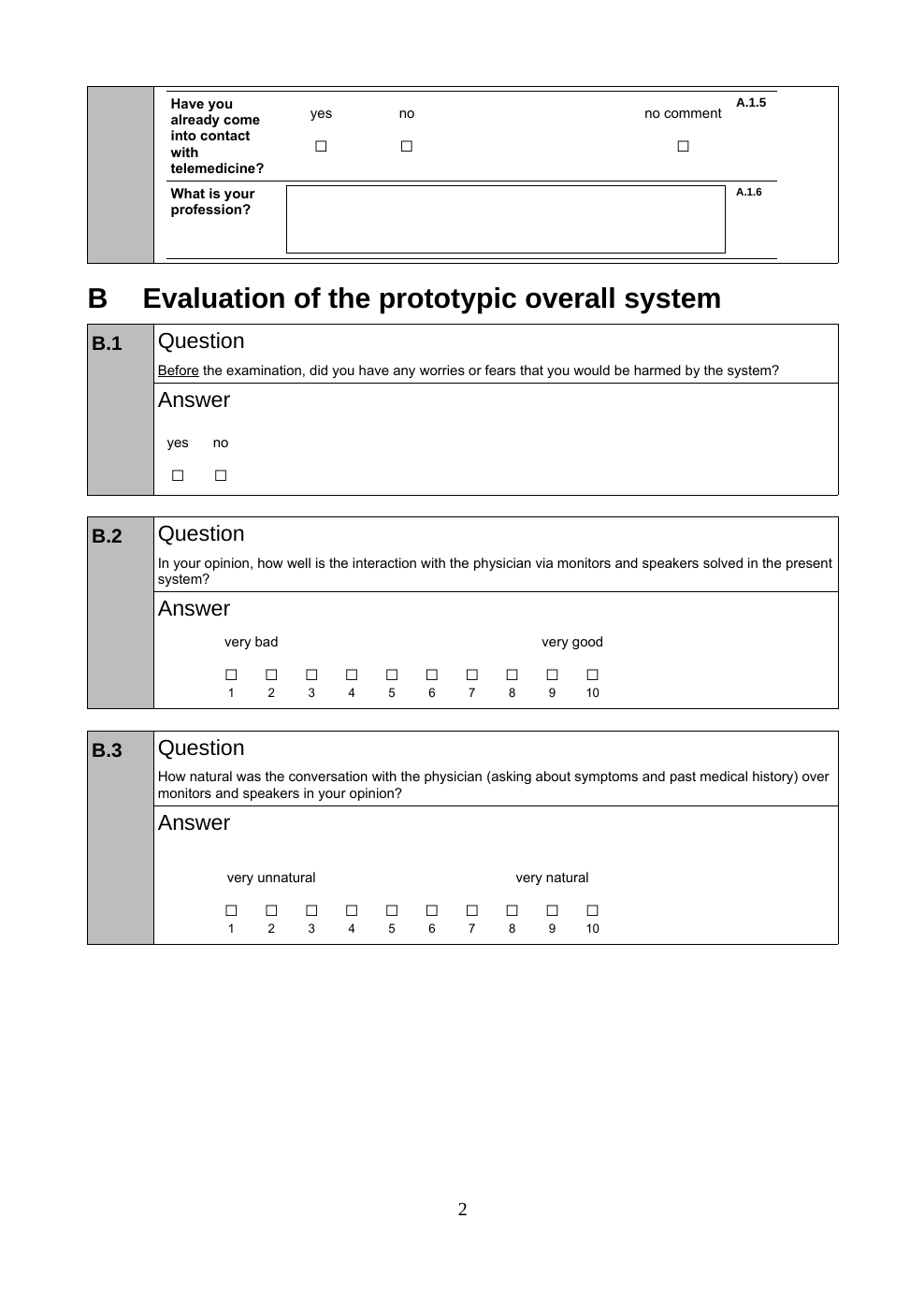


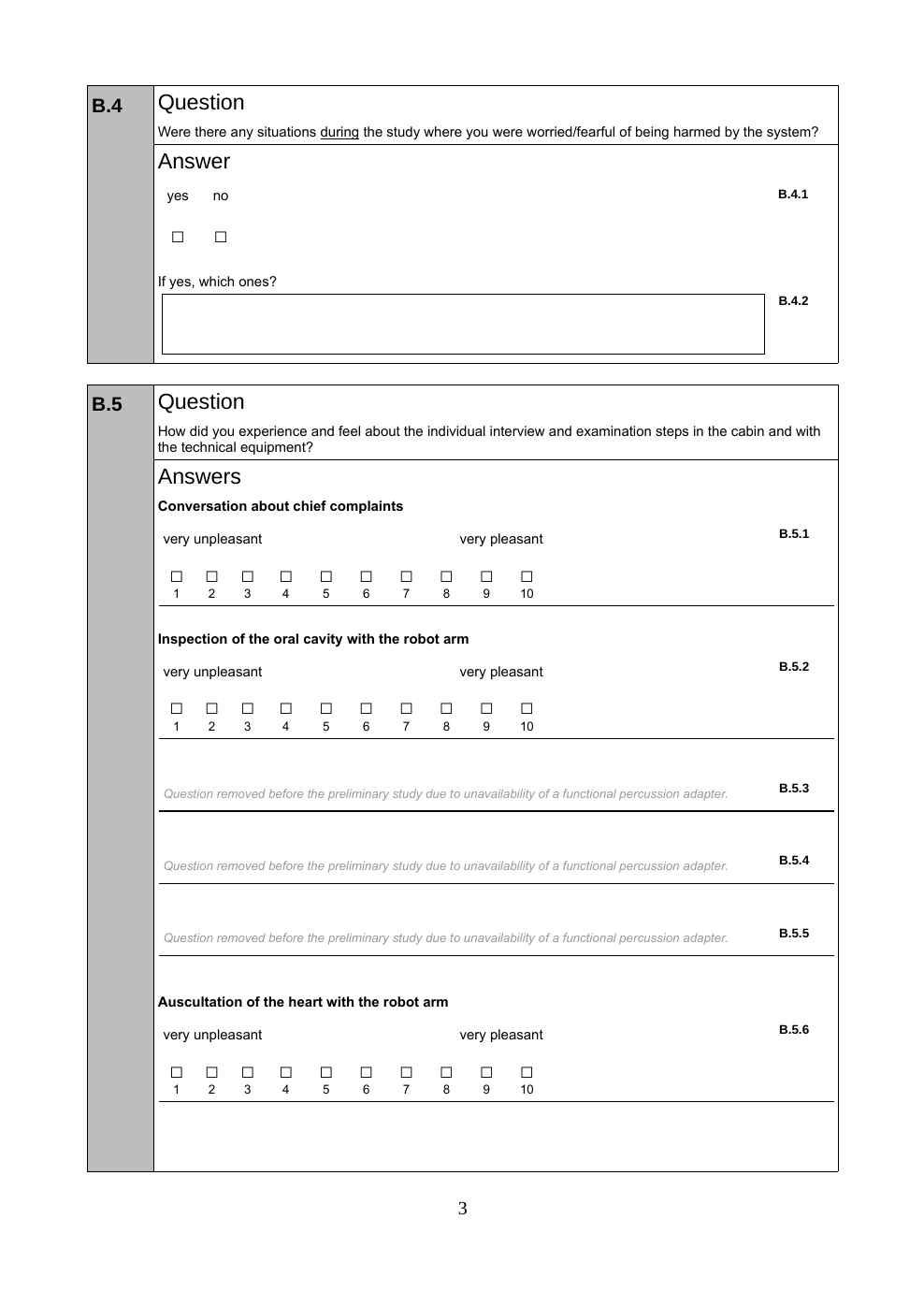


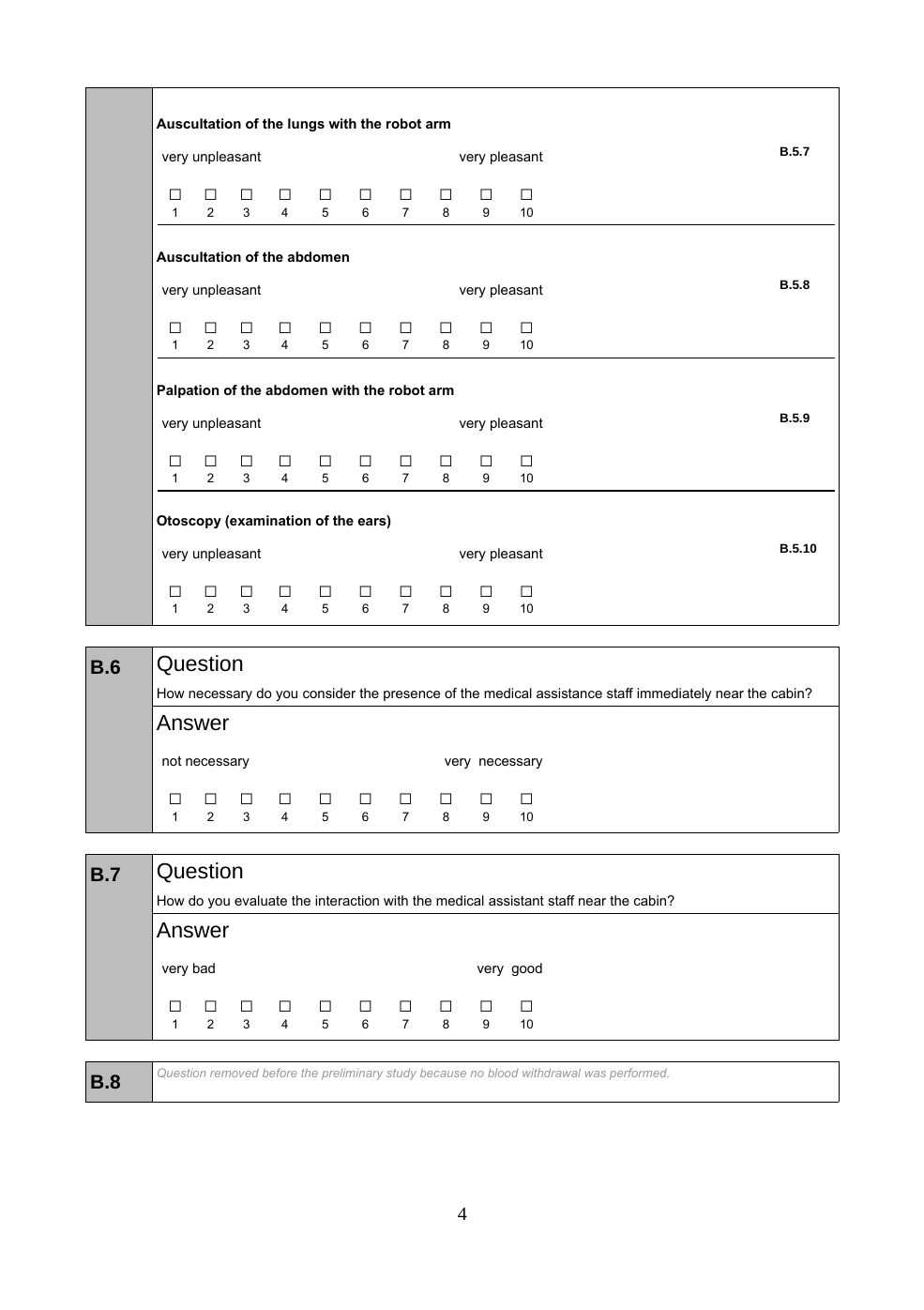


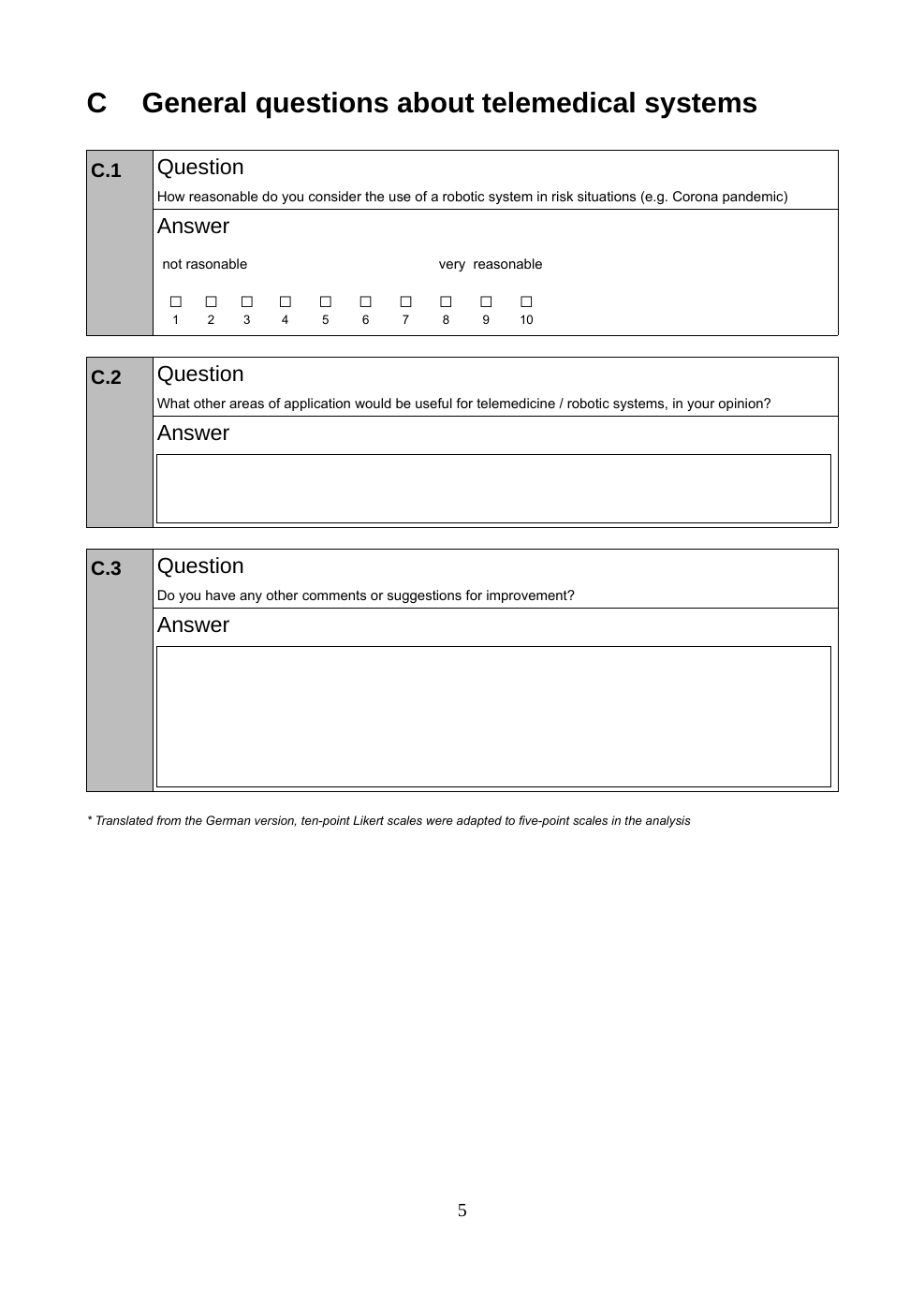


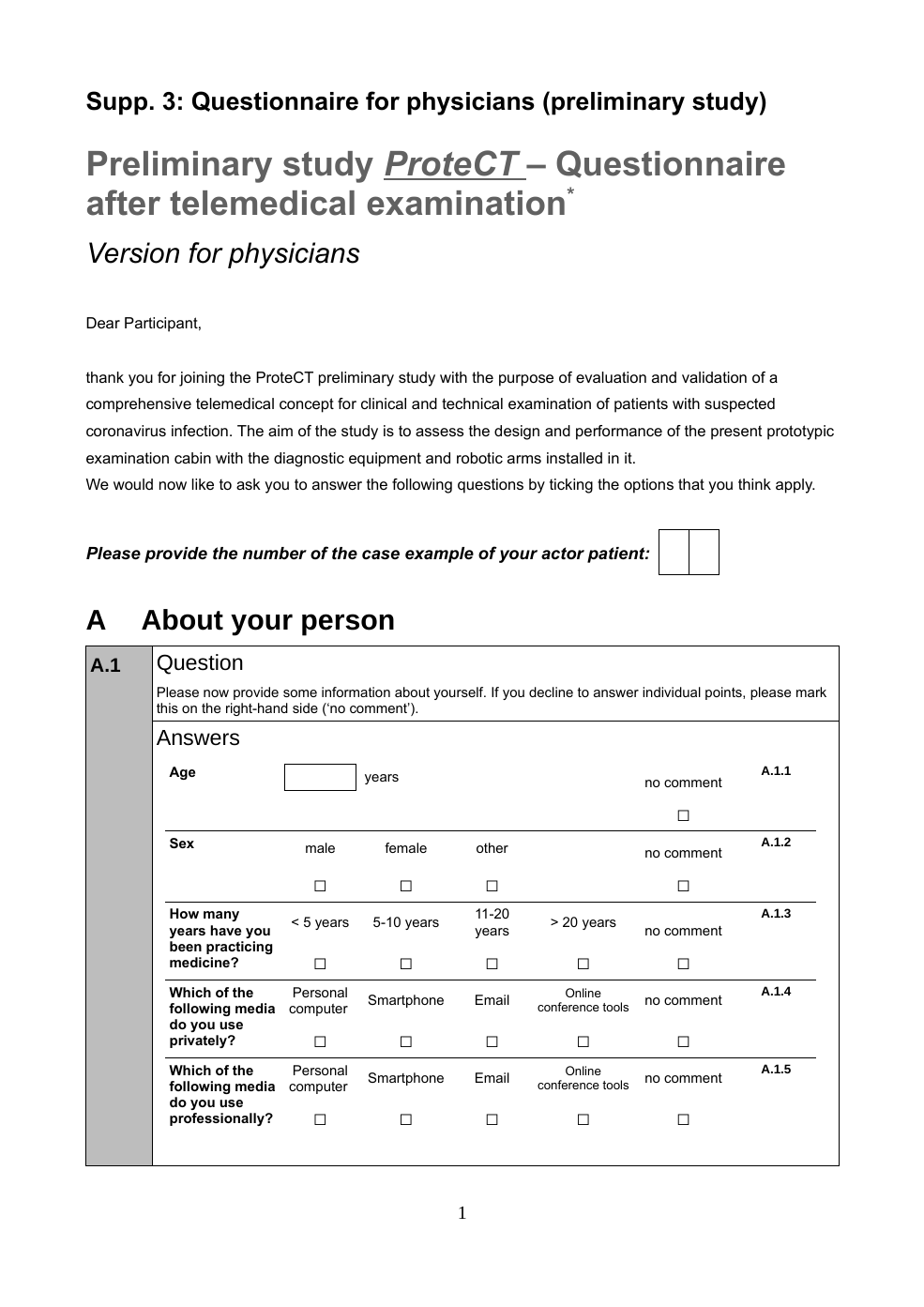


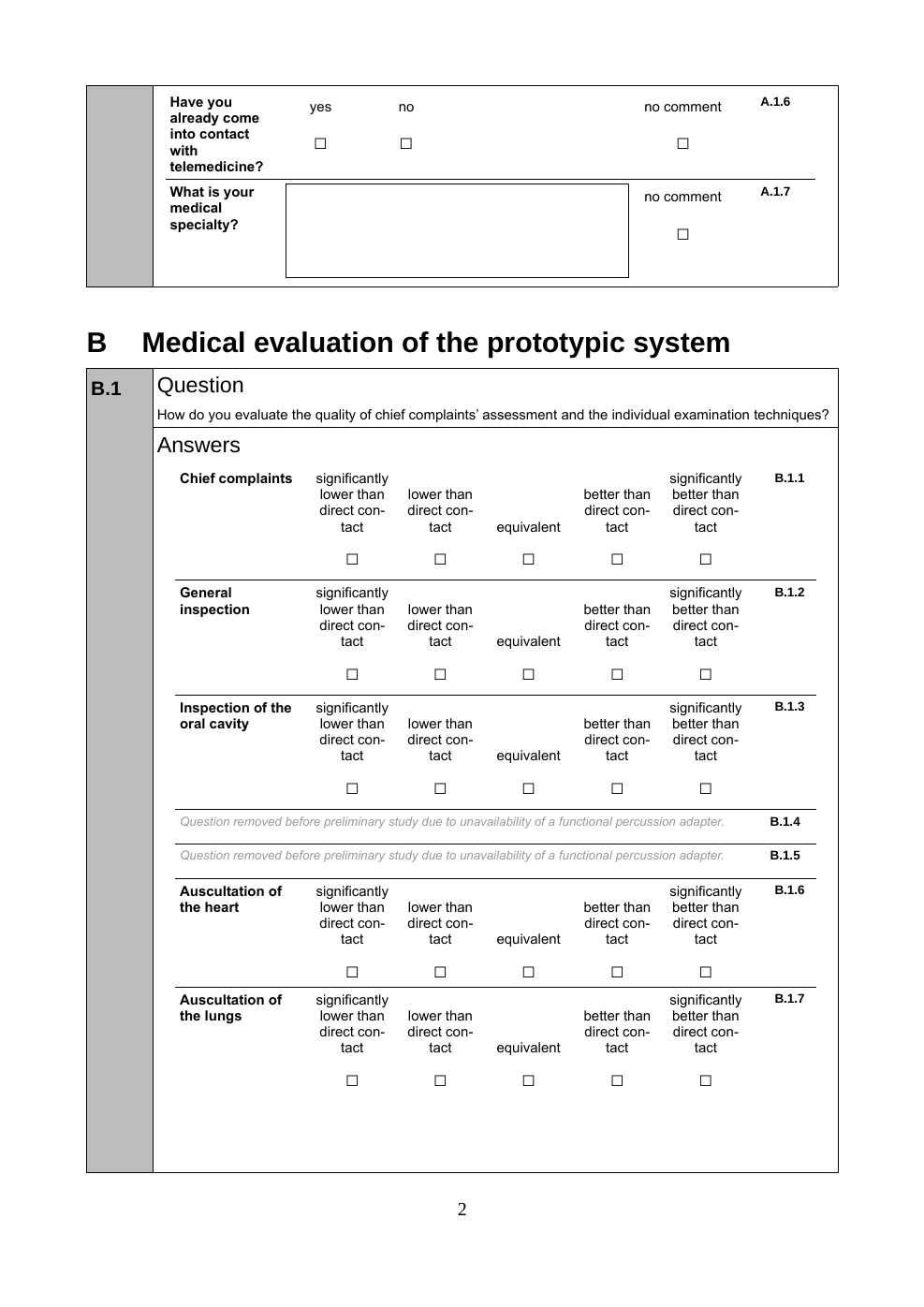


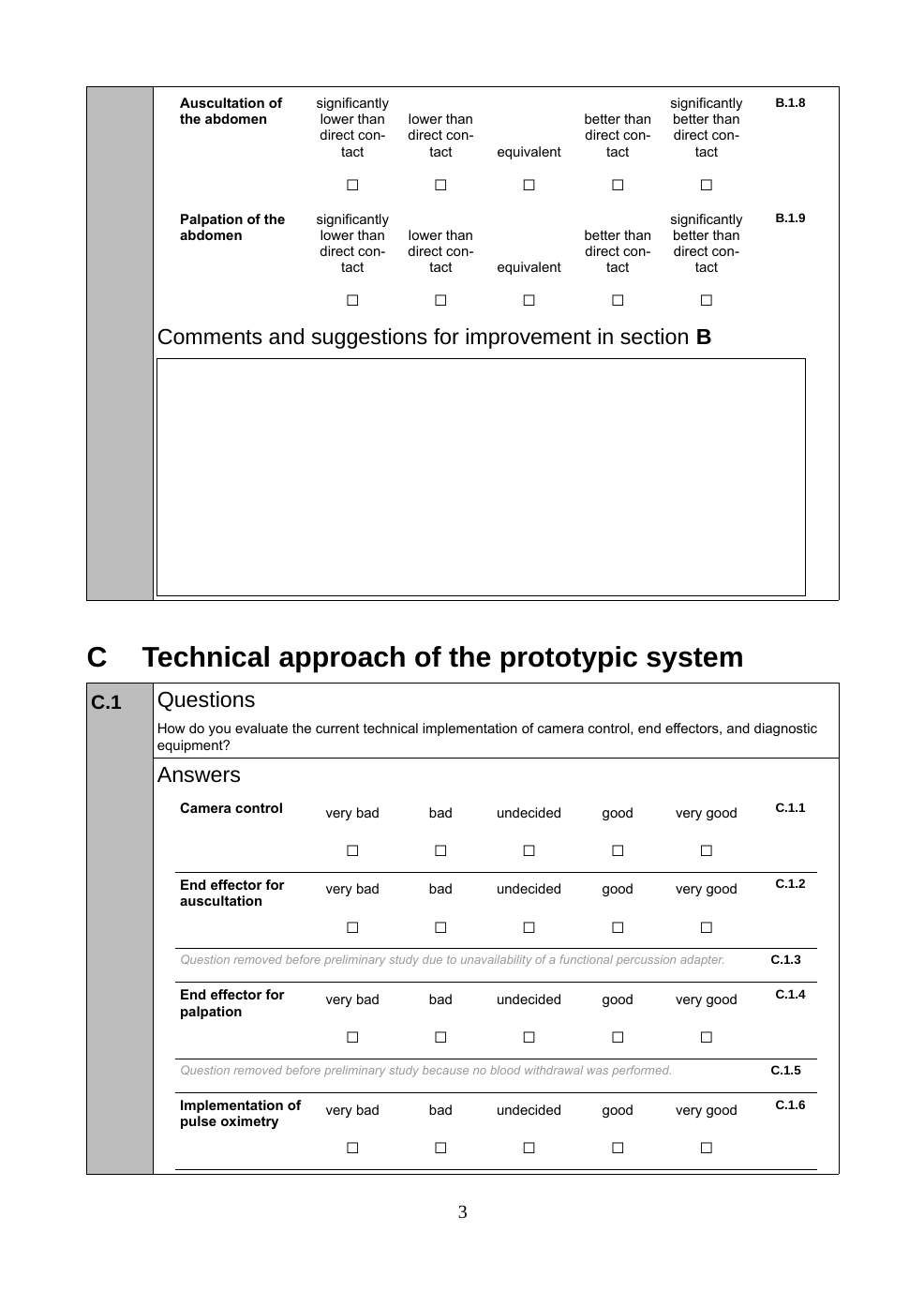


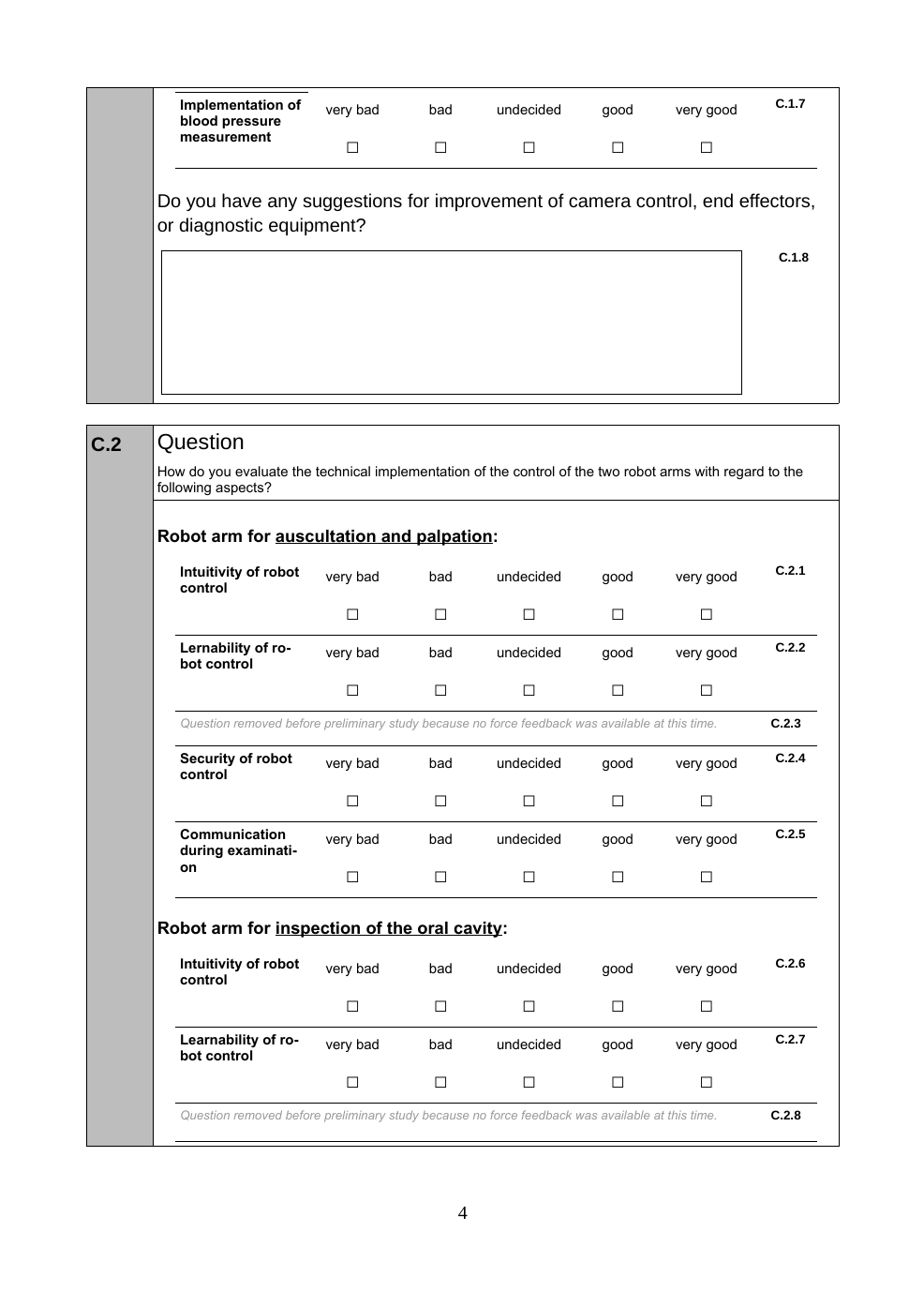


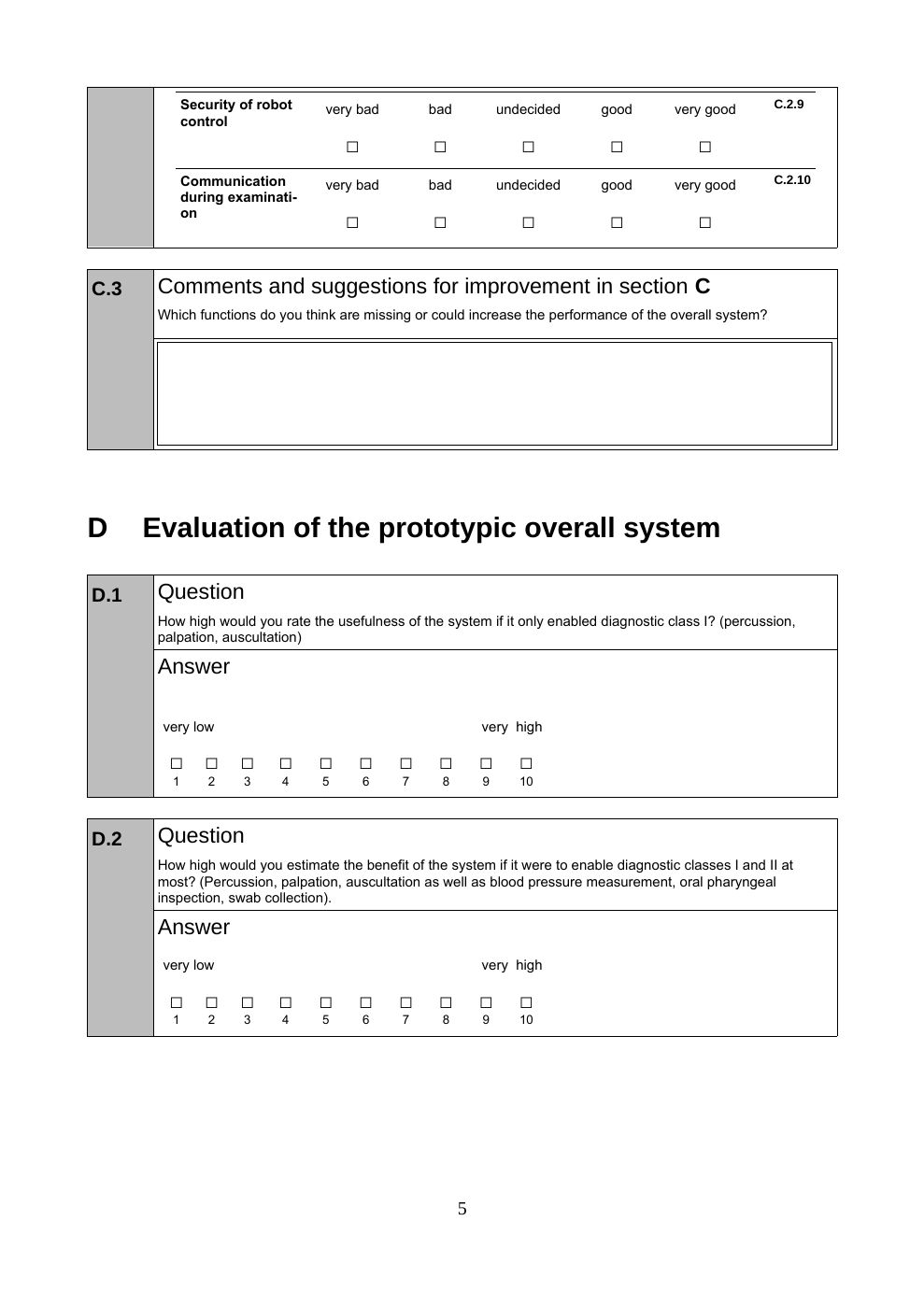


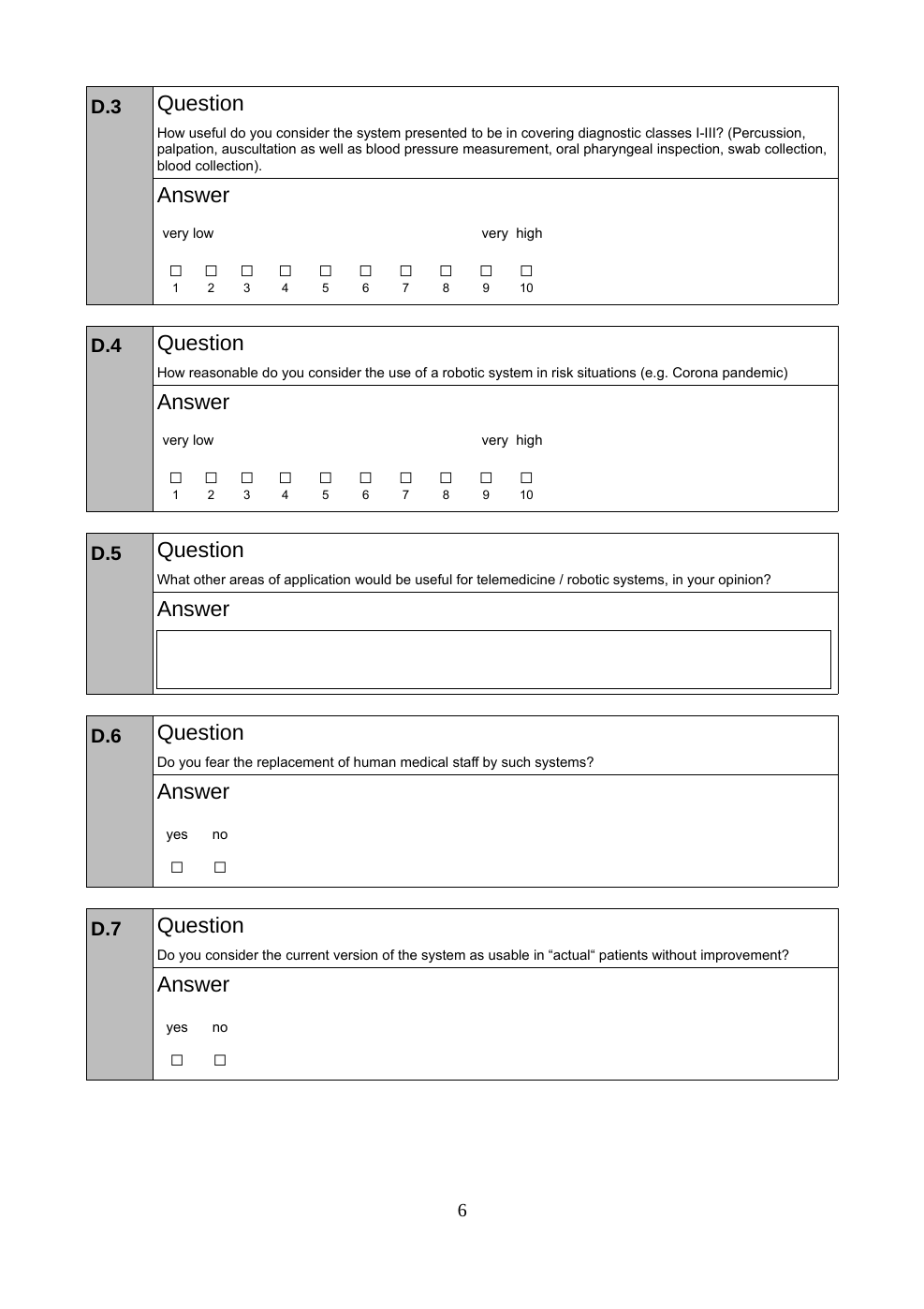


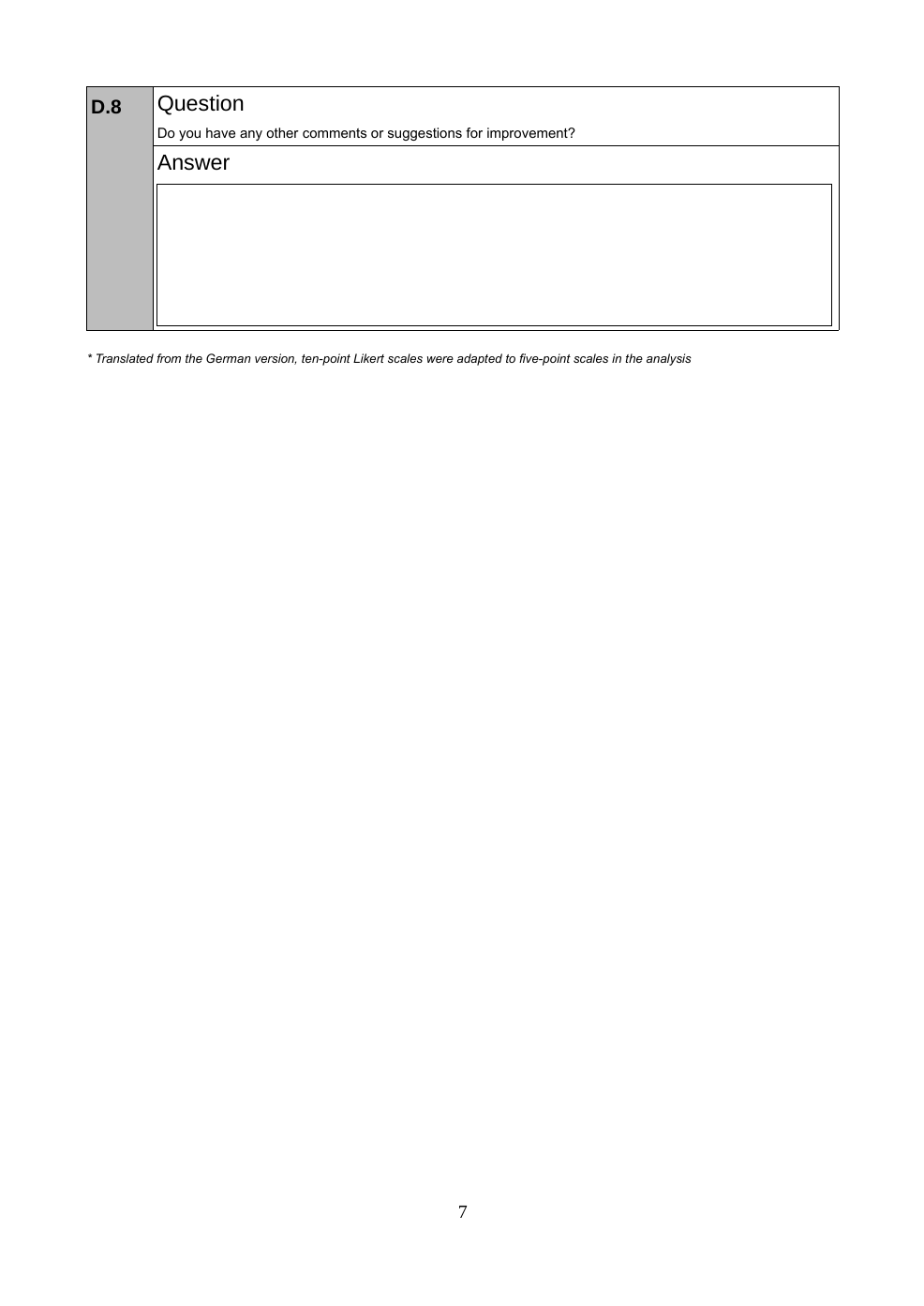


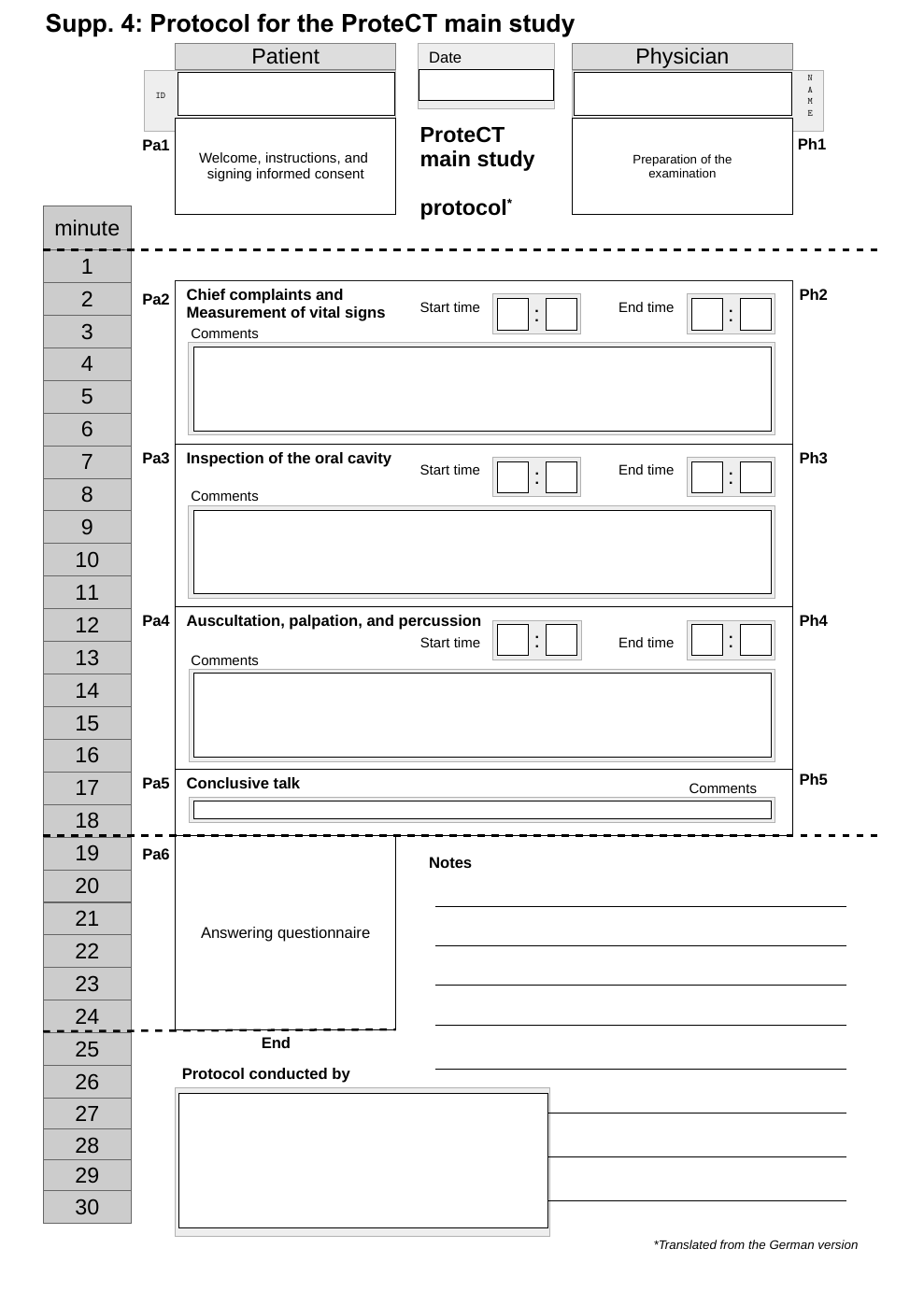


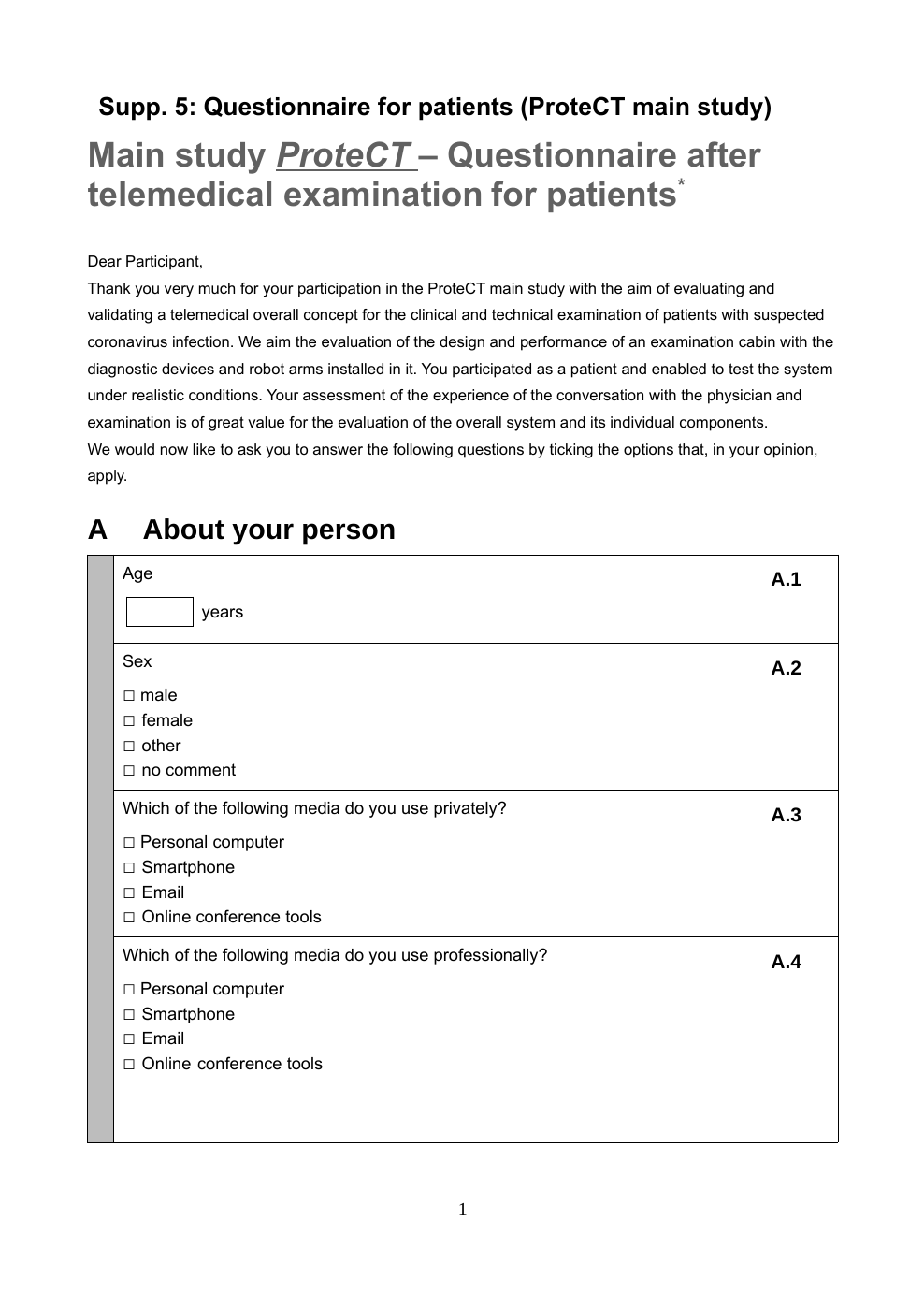


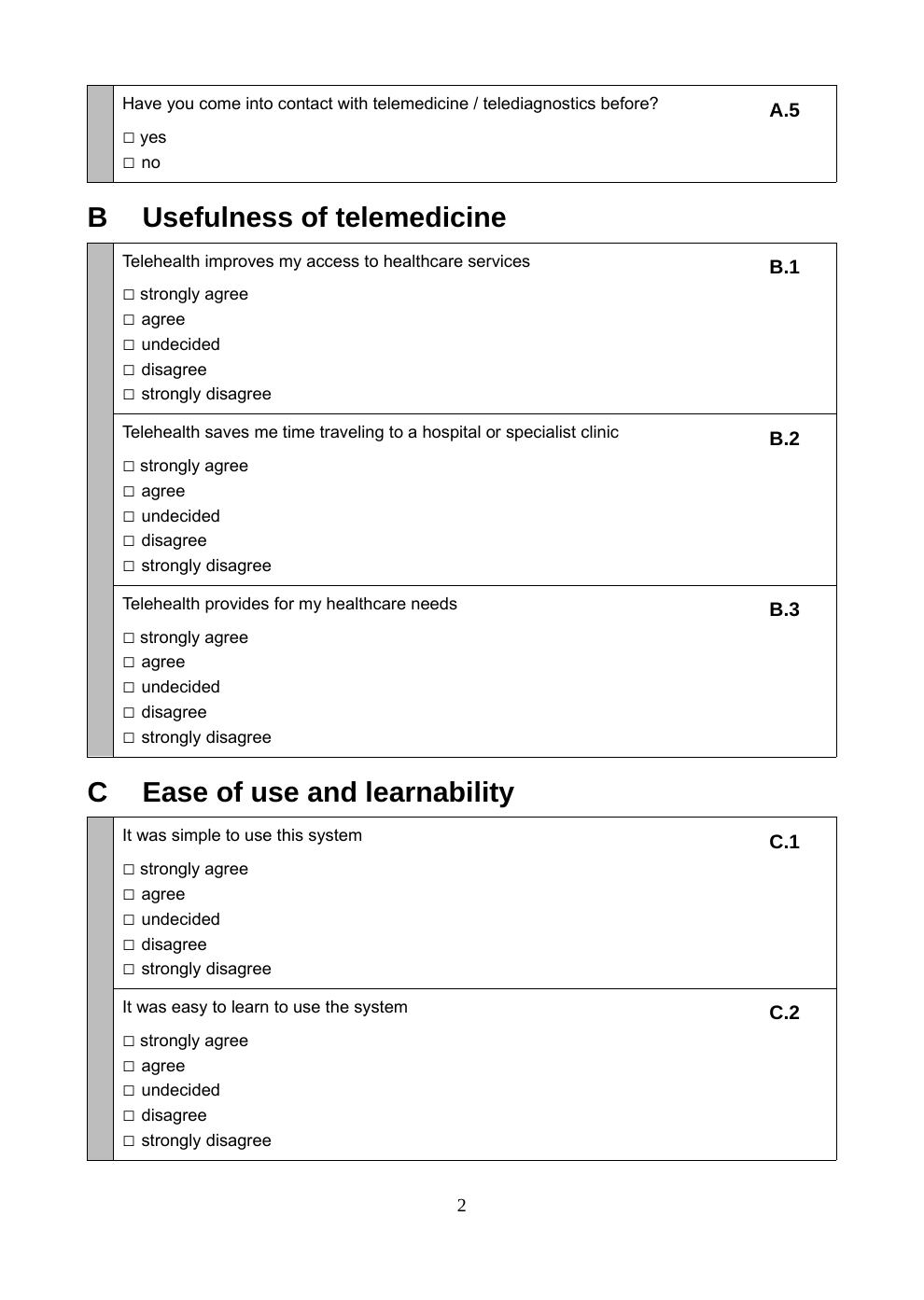


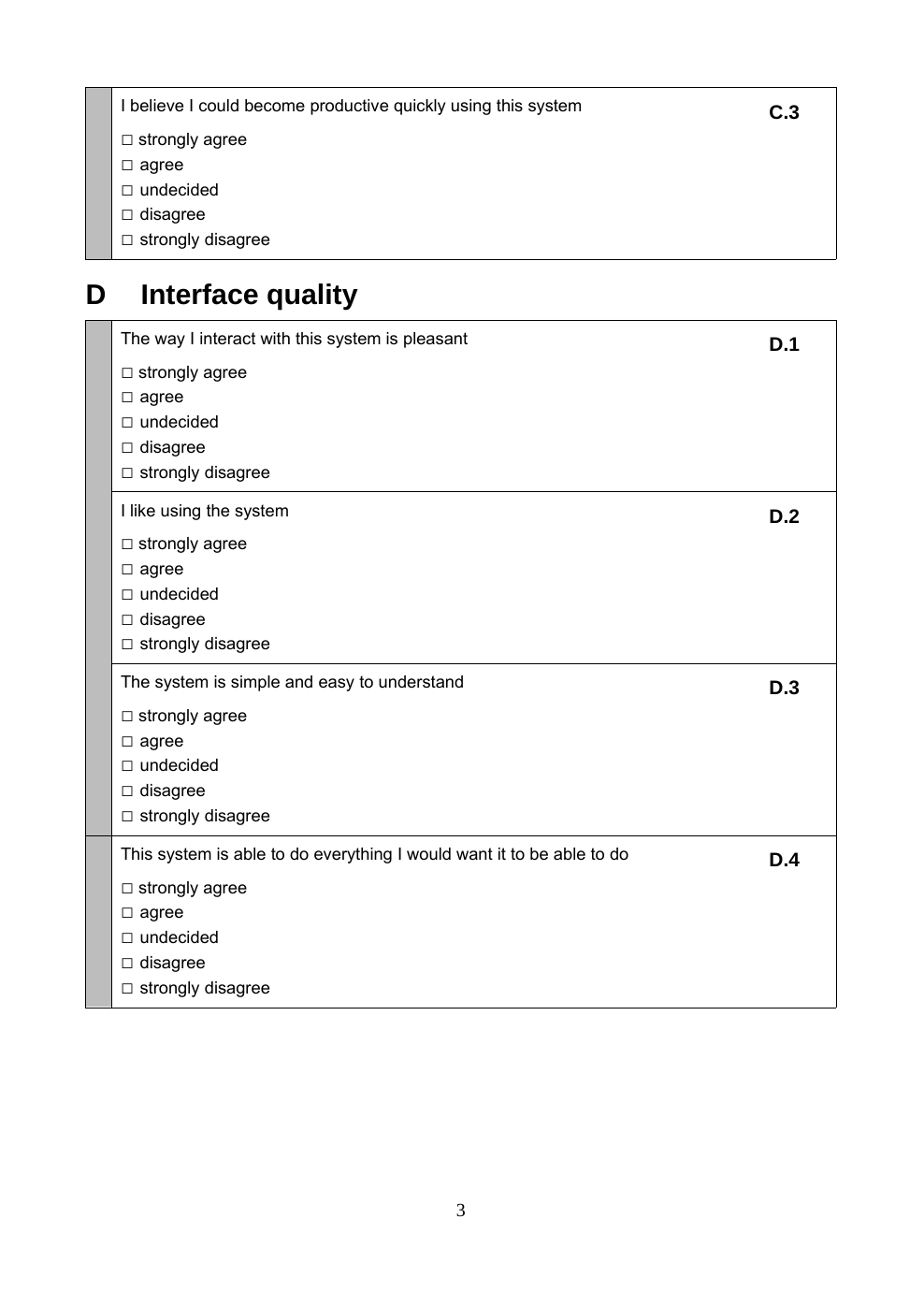


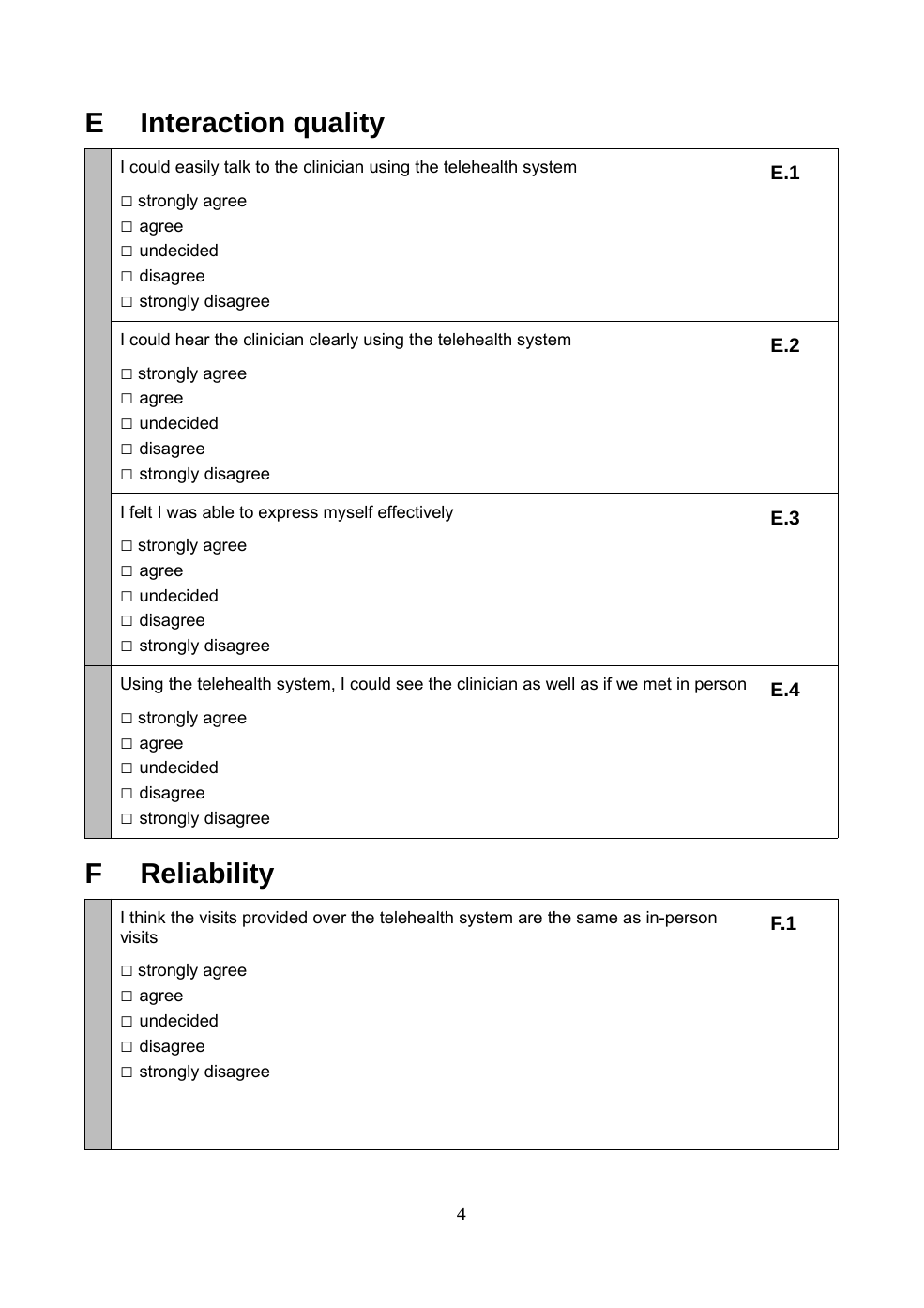


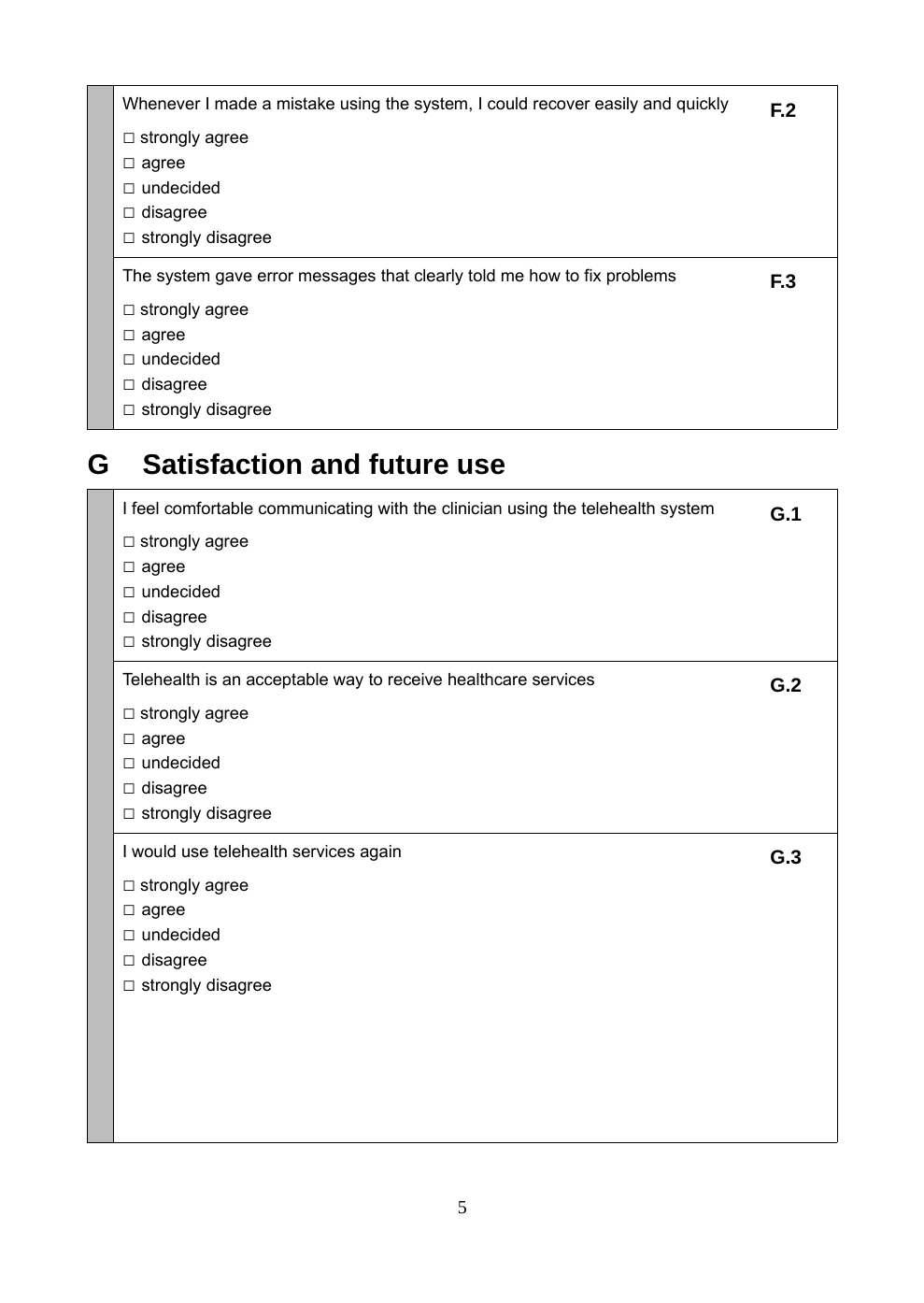


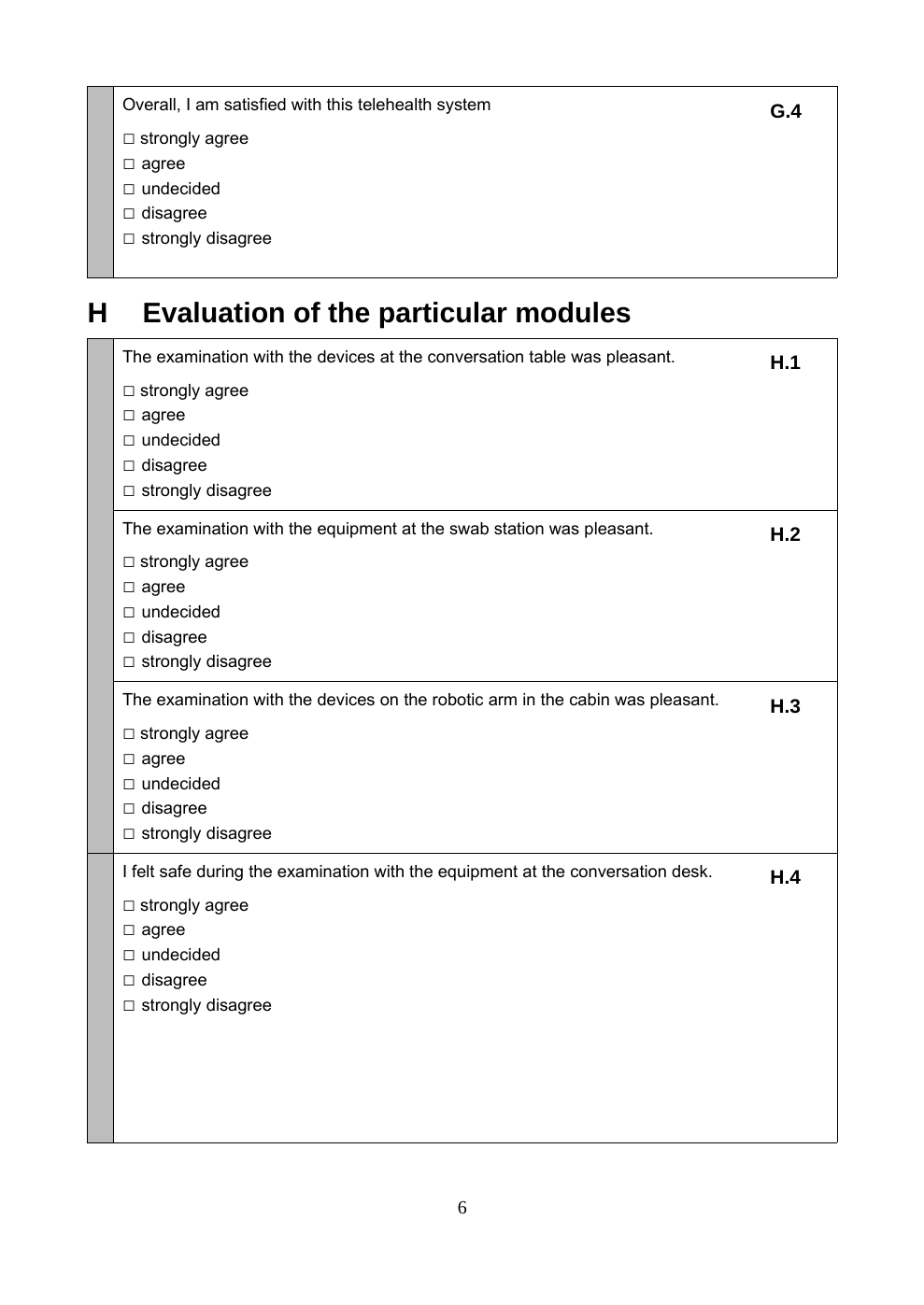


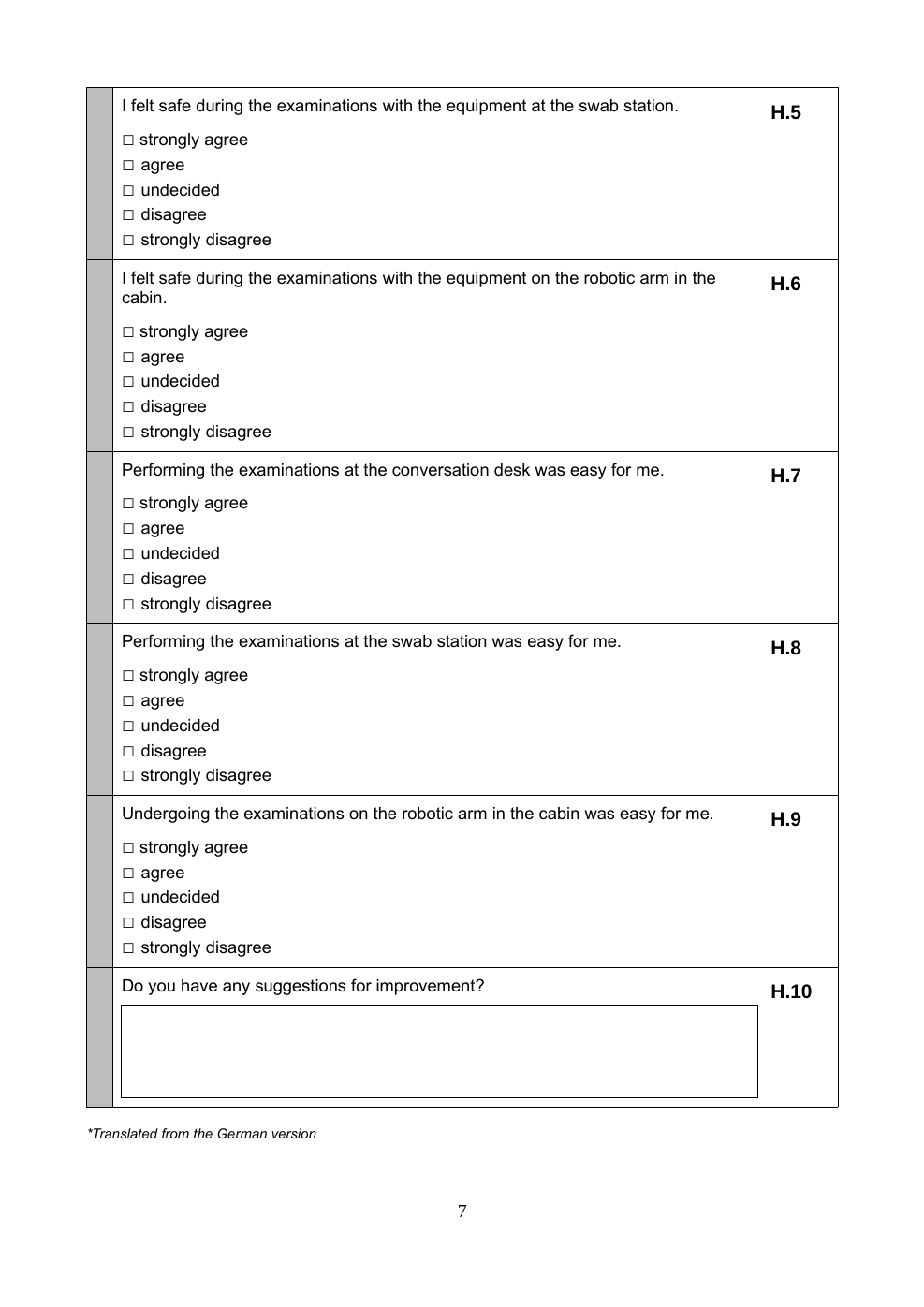


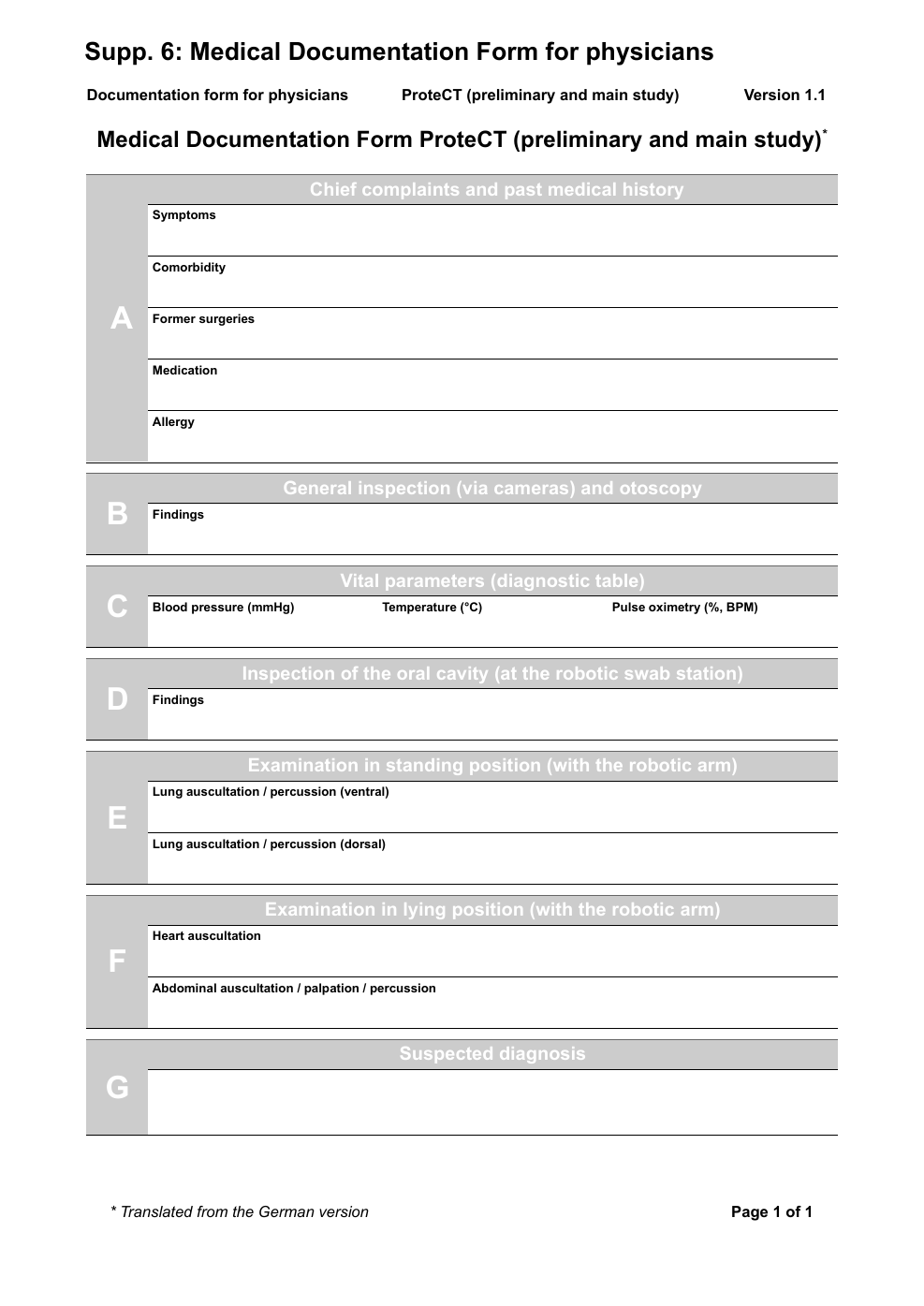


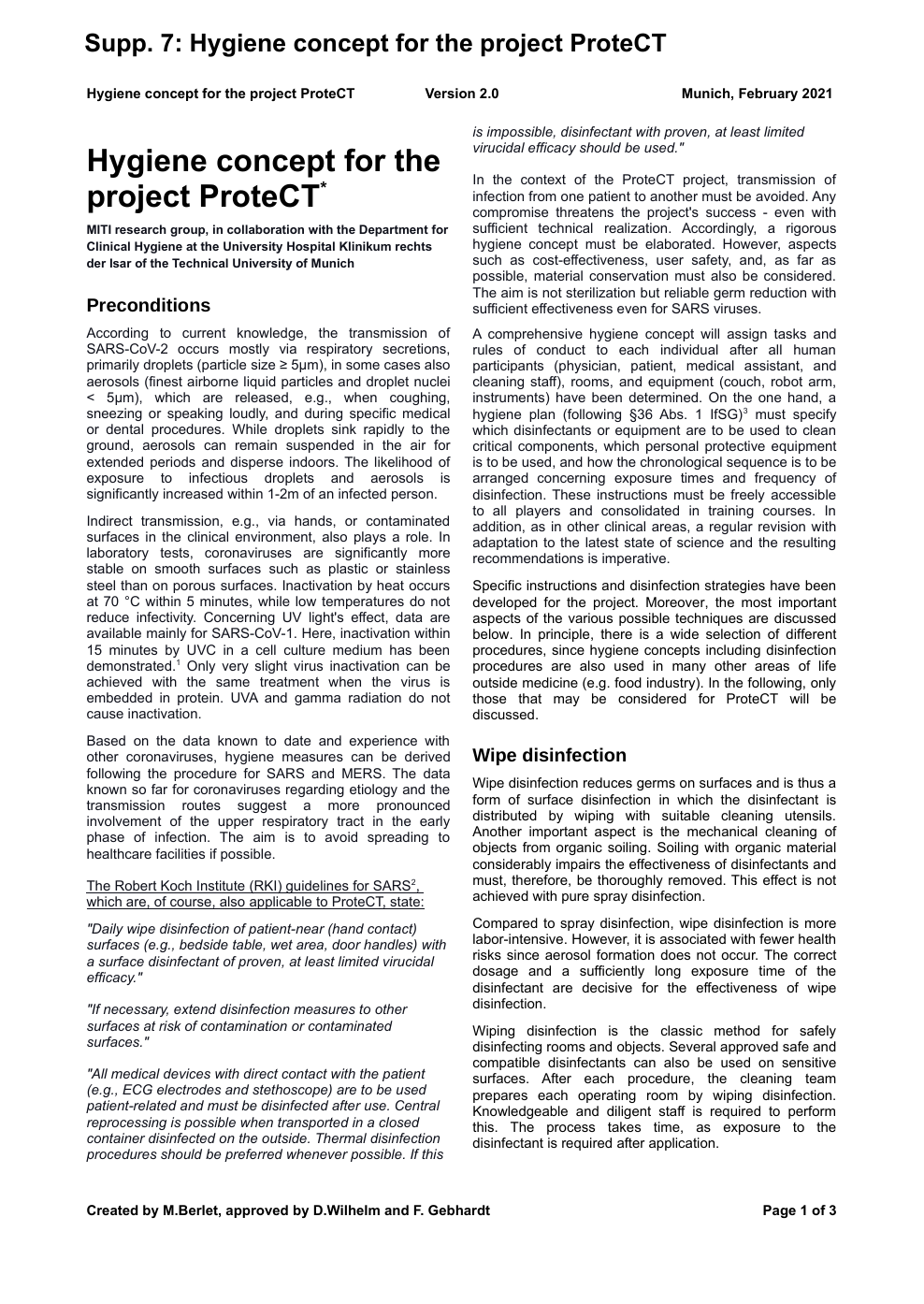


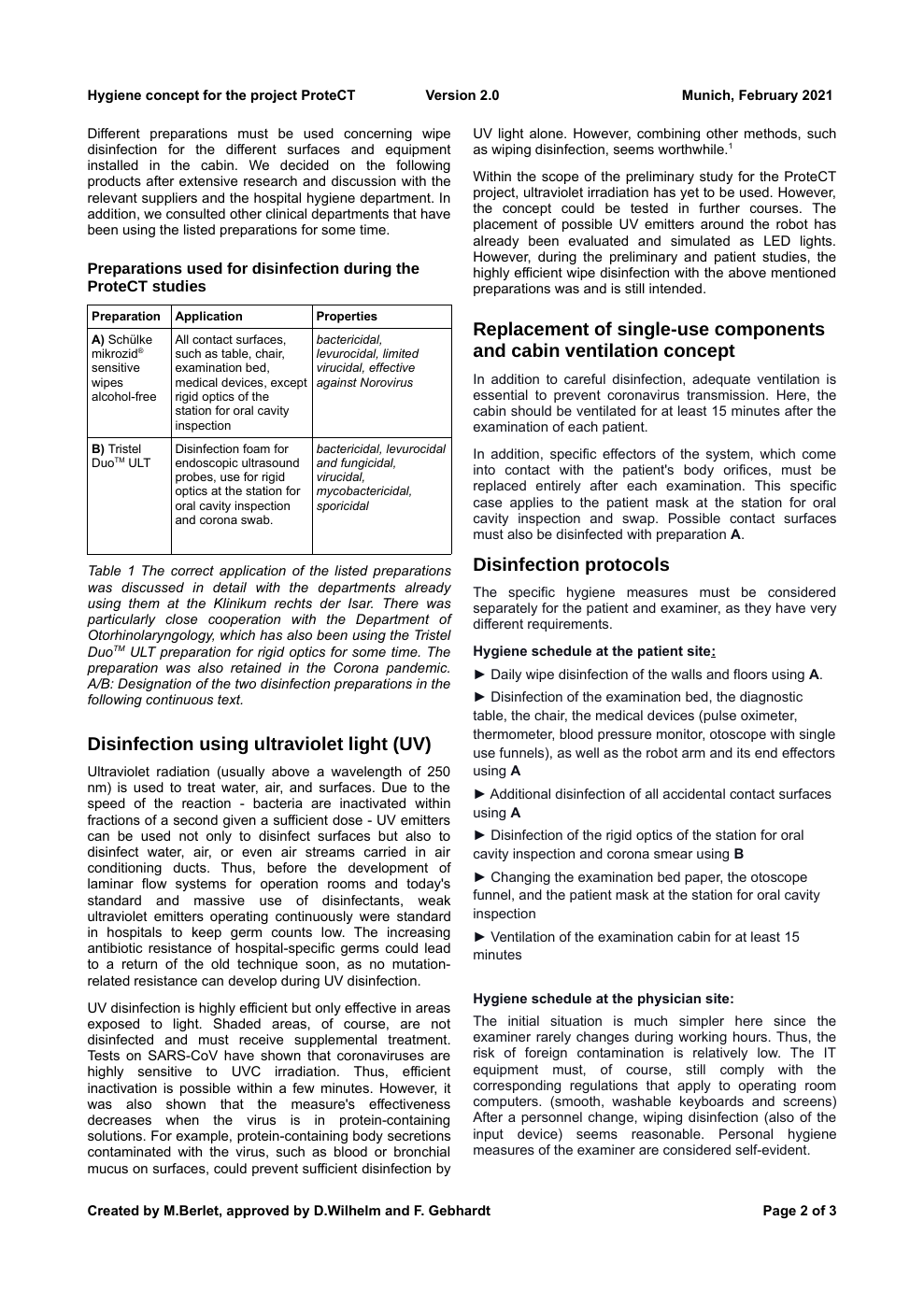


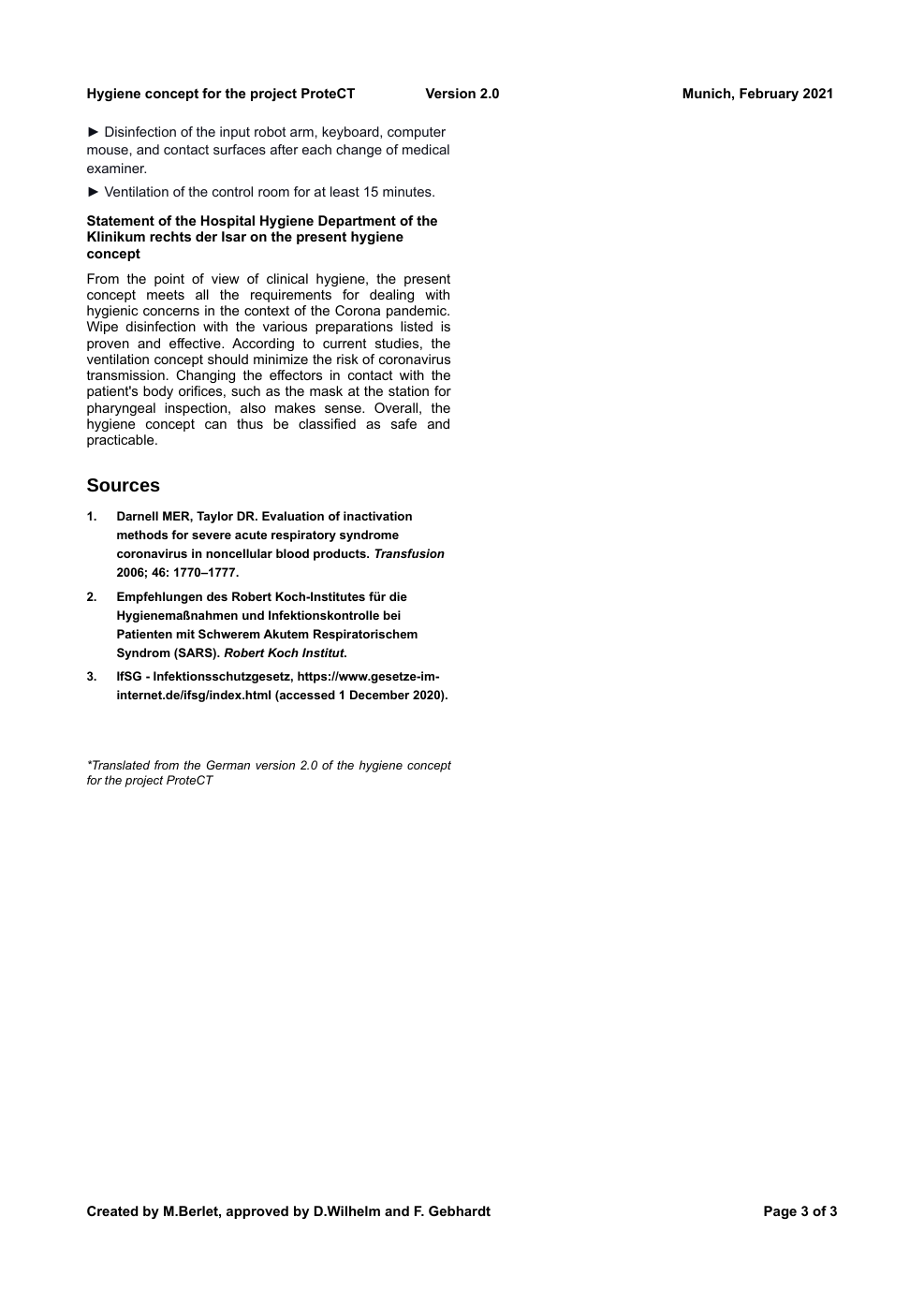


**
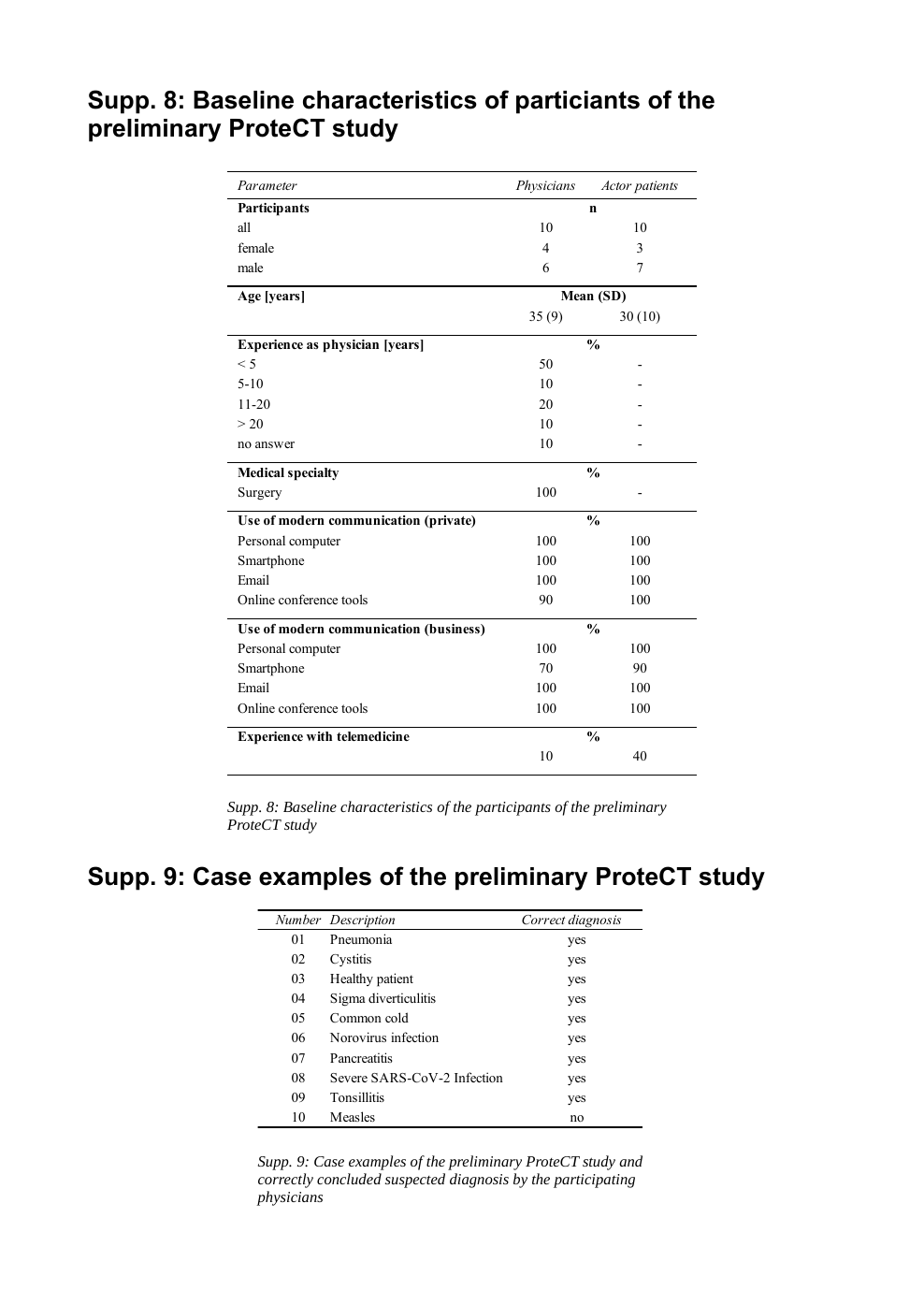
**

**
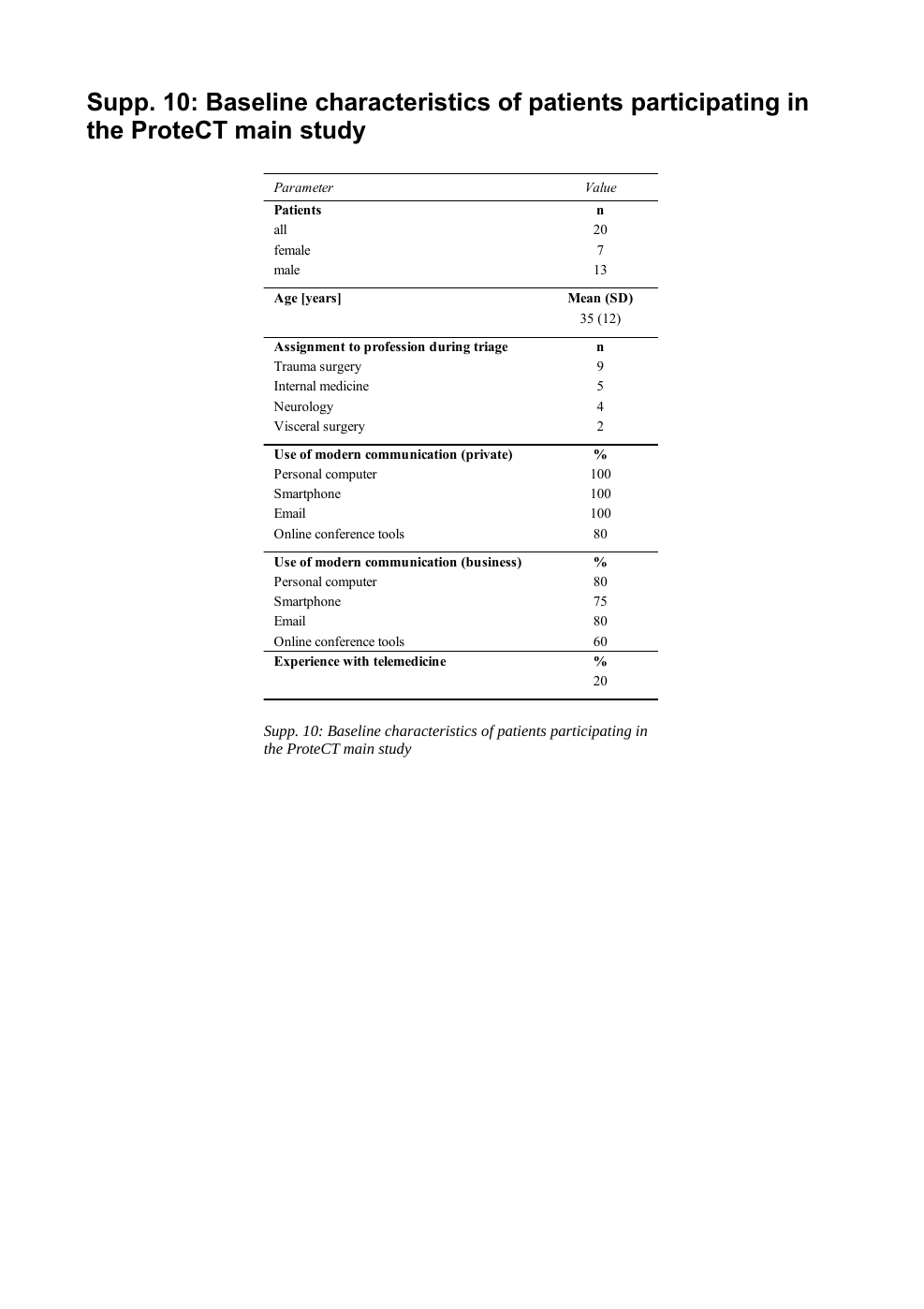
**
